# Near-infrared-featured broadband CO_2_ reduction with water to hydrocarbons by surface plasmon

**DOI:** 10.1038/s41467-023-35860-2

**Published:** 2023-01-14

**Authors:** Canyu Hu, Xing Chen, Jingxiang Low, Yaw-Wen Yang, Hao Li, Di Wu, Shuangming Chen, Jianbo Jin, He Li, Huanxin Ju, Chia-Hsin Wang, Zhou Lu, Ran Long, Li Song, Yujie Xiong

**Affiliations:** 1 Hefei National Research Center for Physical Sciences at the Microscale, School of Chemistry and Materials Science, and National Synchrotron Radiation Laboratory, University of Science and Technology of China, Hefei, 230026 Anhui China; 2 Institute of Energy, Hefei Comprehensive National Science Center, 350 Shushanhu Rd., Hefei, 230031 Anhui China; 3 Institute of Molecular Plus, Tianjin University, 92 Weijin Road, 300072 Tianjin, China; 4 National Synchrotron Radiation Research Center, Hsinchu, 30076 Taiwan; 5 Anhui Engineering Research Center of Carbon Neutrality, College of Chemistry and Materials Science, School of Physics and Electronic Information, and Key Laboratory of Functional Molecular Solids, Ministry of Education, Anhui Normal University, Wuhu, 241002 Anhui China

**Keywords:** Catalytic mechanisms, Photocatalysis, Characterization and analytical techniques, Photocatalysis, Artificial photosynthesis

## Abstract

Imitating the natural photosynthesis to synthesize hydrocarbon fuels represents a viable strategy for solar-to-chemical energy conversion, where utilizing low-energy photons, especially near-infrared photons, has been the ultimate yet challenging aim to further improving conversion efficiency. Plasmonic metals have proven their ability in absorbing low-energy photons, however, it remains an obstacle in effectively coupling this energy into reactant molecules. Here we report the broadband plasmon-induced CO_2_ reduction reaction with water, which achieves a CH_4_ production rate of 0.55 mmol g^−1^ h^−1^ with 100% selectivity to hydrocarbon products under 400 mW cm^−2^ full-spectrum light illumination and an apparent quantum efficiency of 0.38% at 800 nm illumination. We find that the enhanced local electric field plays an irreplaceable role in efficient multiphoton absorption and selective energy transfer for such an excellent light-driven catalytic performance. This work paves the way to the technique for low-energy photon utilization.

## Abstract (web summary)

Changes in Polycomb repression during interphase transition modulate the ability of pluripotent cells to enter cell differentiation.

## Introduction

Artificial photosynthesis offers an appealing way to store intermittent solar energy by directly converting CO_2_, H_2_O and sunlight into hydrocarbon compounds^1–4^. Therewith, a myriad of semiconductors and metal nanocatalysts have been studied, demonstrating their feasibility in light-driven CO_2_ reduction reaction (CO_2_RR)^5–7^. Nonetheless, the low utilization efficiency of the solar spectrum, especially the low-energy photons, and the sluggish process for energy coupling into the reactant molecules are the two critical obstacles that limit their further expansion toward technoeconomic applications. Moreover, the selective production of high-energy-value hydrocarbons involving multiple electron-proton transfer steps through CO_2_RR is another great challenge constraining their potential^8^.

Plasmonic metal nanoparticles exhibit strong interaction with incident light in the form of localized surface plasmon resonance (LSPR)^9,10^. The absorption wavelength center of such LSPR can be easily tuned from ultraviolet (UV) to near-infrared (NIR) range just by changing the geometry structure (including symmetry, size, etc.) of plasmonic nanomaterials^11,12^, allowing them to have the wide possibility as light-harvesting units for solar-to-chemical energy conversion^13–19^. Recently, it was demonstrated that specific plasmonic nanostructures are able to convert CO_2_ under laser irradiation with the help of organic additives^20,21^, suggesting their potential in CO_2_RR. In addition, the rates of plasmon-induced catalytic reaction exhibit a super-linear power law dependence on the number of incident photons, providing a positive relationship between quantum efficiency and photon flux^22^. Amidst the fascinating development of such plasmonic materials, it is discovered that the mismatch of quick LSPR relaxation (up to ~100 ps) and slow chemical reduction kinetics (several milliseconds or seconds) in time scale severely retards the conversion efficiency of solar energy^23–25^. To break this bottleneck, versatile co-catalysts are employed to establish an effective bridge for transferring hot carriers from plasmonic materials into reactant molecules^13–17,26^.

In this respect, the precise design of the co-catalyst toward efficient hot carrier accepting ability and unique CO_2_ chemisorption capability is highly sought. Here we employ Au rod as a plasmonic light-harvesting unit and copper-palladium (CuPd) alloy shell as a co-catalyst to realize efficient plasmon-induced artificial photosynthesis in the absence of the additional sacrificial agent, achieving nearly 100% CH_4_ product selectivity under full-spectrum light illumination. As deciphered by in situ near ambient pressure X-ray photoelectron spectroscopy (NAP-XPS) and density functional theory (DFT) calculations, the localized electric field, which leads to the emergence of a new isolated state above the Fermi energy (*E*_f_) and the differentiation of electron transfer within different molecular orbitals, enables such a unique solar-to-chemical energy conversion. By optimizing the reaction system, an impressive CH_4_ production rate of 0.55 mmol g^−1^ h^−1^ is achieved via a gas-solid biphase system, demonstrating a record-high apparent quantum efficiency (AQE) of 0.38% at 800 nm NIR light illumination.

## Results

### Plasmon-induced CO_2_ reduction with water

**Fig. 1:**
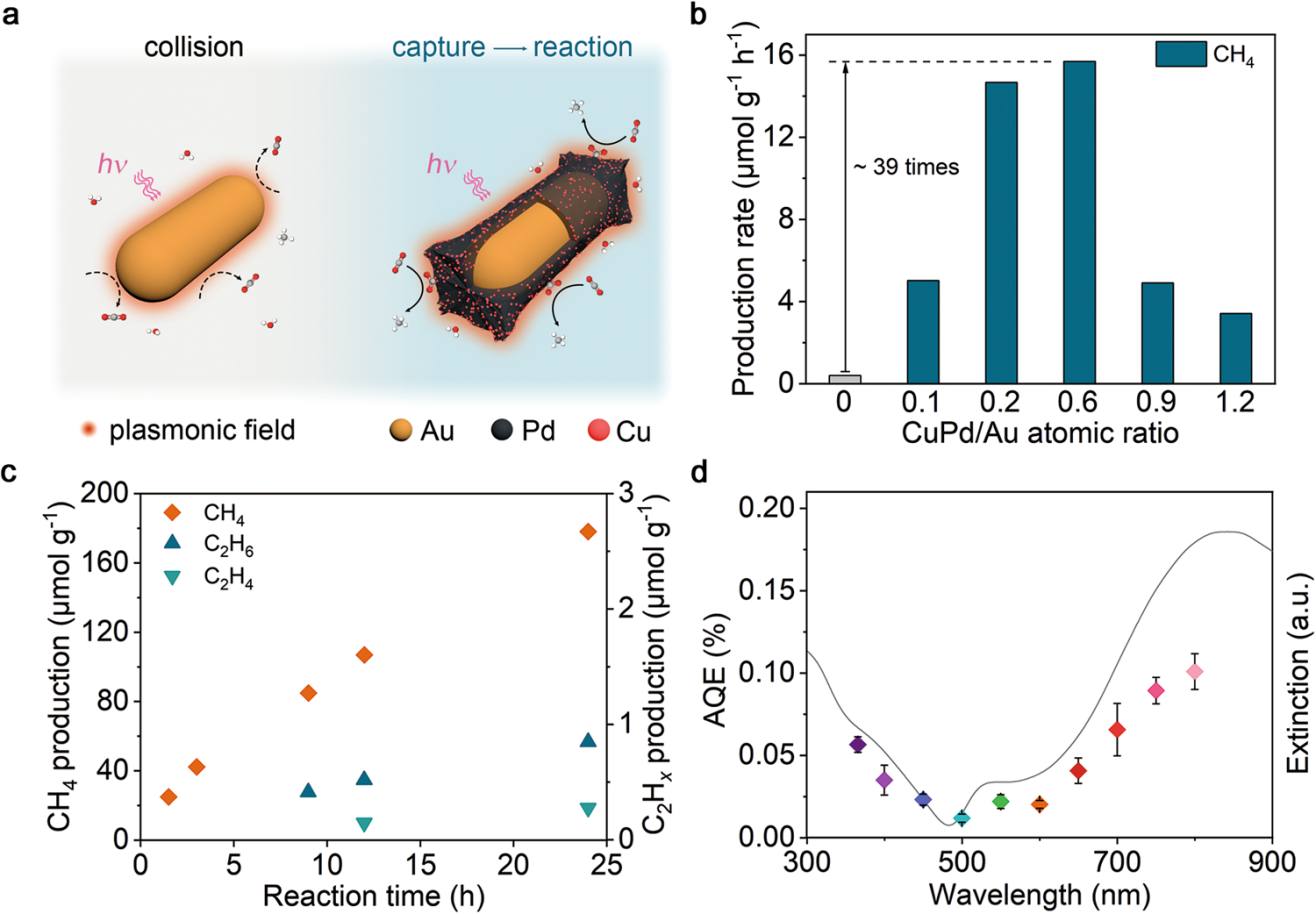
Plasmon-induced CO_2_RR performance. **a** Schematic illustration of the role of CuPd co-catalyst in capturing CO_2_ molecules. Plasmonic catalysis usually takes place very close to the catalyst surface (i.e., within the range of the plasmon-induced local field). For the pure Au nanorods, the probability of the pure Au nanorods and CO_2_ molecules to contact through the collision is very low (left), resulting in low CO_2_ conversion efficiency. For the Au rod@CuPd, CuPd co-catalyst can capture CO_2_ molecules and enhance the CO_2_ concentration on the catalyst surface, increasing the opportunity for their further activation and conversion (right). **b** Average production rates of CH_4_ over Au rod@CuPd_2_ with different shell thicknesses. **c** Time-dependent CH_4_ production over Au rod@CuPd_2_ in 24 h. **d** Calculated AQEs (color dots) over Au rod@CuPd_2_ under different monochromatic light illumination, in reference to its UV-vis extinction spectrum (black line). The error bars represent the standard deviation of the experiments. The catalytic experiments of (**b**,**c**) are all carried out in CO_2_-saturated water and CO_2_ atmosphere without any sacrificial agent under 400 mW cm^**–**2^ full-spectrum light illumination.

The CuPd co-catalyst can capture CO_2_ molecules and enhance the CO_2_ density on the catalyst surface, which allows the reactants to locate within the range of the plasmon-induced local field, increasing the opportunity for their further activation and conversion (see Fig. 1a). Typically, we obtain the Au rod@CuPd core-shell composites via a wet chemical synthesis (Fig. S1). The shell thickness and Pd/Cu ratio of the Au rod@CuPd core-shell composites can be easily controlled by changing the amount of metal precursors (see Tables S1, S2, and Figs. S2–S5 for the list of prepared samples). Taking Au rod@CuPd_2_ as an example, the transmission electron microscopy (TEM) image (Fig. S3a) shows that the rod structure is preserved after the deposition of the CuPd shell onto the Au surface. The fast Fourier transform patterns and magnified images of the selected area in Fig. S6, a,b, are well-fitted with a pure face-centered cubic crystal structure viewed along the [110] and [100] directions, respectively. The ordered lattice fringes with a spacing of 2.35 and 2.05 Å are attributed to the (111) and (002) planes of Au lattice, respectively, indicating that precursor ions are epitaxially reduced on the surface of Au rods to form a CuPd single-crystal shell. Such a result can be also supported by the X-ray diffraction (XRD) analysis (Fig. S7) and the elemental mapping profiles (Fig. S8). In addition, the Pd and Cu K-edge X-ray absorption near-edge structure (XANES, Fig. S9) and Fourier transformed extended X-ray absorption fine structure (EXAFS, Fig. S10) spectra of the prepared samples show that the total coordination numbers of Pd and Cu are smaller than 12 (see Table S3), further confirming that the Pd and Cu elements are located in the outermost shell of these samples. Then we employ UV-vis extinction spectra to evaluate the light-harvesting ability of the prepared samples. As shown in Fig. S11, two peaks around 510 and 740 nm are the typical characteristics of the transversal and longitudinal LSPR modes of Au nanorod, respectively^27,28^. It is discovered that, compared with the Cu/Pd molar ratio, the CuPd thickness has a more prominent influence on the optical properties, allowing the remarkable broadening of the absorption range of prepared samples into the NIR region.

Upon having a comprehensive understanding of the structural information and optical properties of the Au rod@CuPd, we assess their efficacy for CO_2_RR in a conventional reactor consisting of CO_2_-saturated water and suspended catalysts (see Fig. S12). After continuous illumination for 3 h, only CH_4_ can be detected with a maximum production rate of 15.6 μmol g^−1^ h^−1^, ca. 39 times that of Au rod, demonstrating the excellent catalytic performance of Au rod@CuPd_2_ (Fig. 1b). The production rates of CH_4_ show a volcano-shaped relationship with the CuPd/Au ratios, which can be attributed to the competition of increased surface active sites and aggravated electron-phonon scattering in the thickened CuPd shell^16^. The linear time-dependent CH_4_ production shown in Fig. 1c corroborates the excellent durability of Au rod@CuPd_2_. Moreover, C_2_H_6_ and C_2_H_4_ can also be detected after the long-term test, suggesting the presence of multiple proton-coupled electron transfer processes during the reaction, which is uncommon in conventional photocatalysis. In the meantime, after normalizing the CH_4_ production rates by the amount of Cu (orange dots in Fig. S13), the catalytic performances of samples with different Cu/Pd atomic ratios are similar, suggesting the inseparable role of Cu in CO_2_RR. The wavelength-dependent apparent quantum efficiencies (AQEs), which are in good agreement with the extinction spectrum of Au rod@CuPd_2_, are up to 0.1% at 800 nm (see Fig. 1d). This result explicitly suggests the unique features of Au rod@CuPd_2_ in effectively utilizing low-energy photons in the absence of the sacrificial agent (see also Fig. S14). In cyclic tests under 800 nm monochromatic light illumination, the production rates of CH_4_ are well maintained for 10 runs (each run for 3 h, Fig. S15), verifying the long-term stability of Au rod@CuPd_2_. Furthermore, hydroxyl radicals (**•**OH) are detected as the main oxidative product by electron spin resonance spectroscopy (Fig. S16).

The verification of product origin is another key aspect. The conditions for various control experiments and corresponding results are illustrated in Fig. S17 (see also Table S4 for details). Unambiguously, catalyst, light, CO_2_ and H_2_O are keys for CO_2_RR over Au rod@CuPd_2_ and the CH_4_ can hardly be produced from the direct photolysis or pyrolysis of the carboneous compounds (e.g., surfactants and reducing agents for catalyst synthesis). We further employ synchrotron radiation vacuum UV photoionization mass spectrometry (SVUV-PIMS) to convincingly identify the origin of the CH_4_ product. As shown in Fig. S18, a,b, the peaks at m/z = 16 and 17 can be assigned to ^12^CH_4_ and ^13^CH_4_ when using ^12^CO_2_ and ^13^CO_2_ as the feed gas, respectively, firmly suggesting that the CO_2_ feed gas is indeed the carbon source of produced CH_4_. In addition, we employ offline sum frequency generation vibrational spectroscopy (SFG-VS) to monitor the surface intermediates formed during the photocatalytic reaction. As shown in Fig. S19, a,b, after illumination for 5 min, three SFG-VS signals appear at 2880, 2945, and 2965 cm^−1^, which can be assigned to the symmetric stretching (ν_s_ C–H), Fermi-resonance (ν_Fermi_ C–H) and asymmetric stretching (ν_as_ C–H) modes of the generated methyl groups, respectively^29^. This observation firmly demonstrates the evolution of CO_2_ and H_2_O to methyl groups at the catalytic interface.

### Mechanism for plasmon-induced catalytic process

**Fig. 2:**
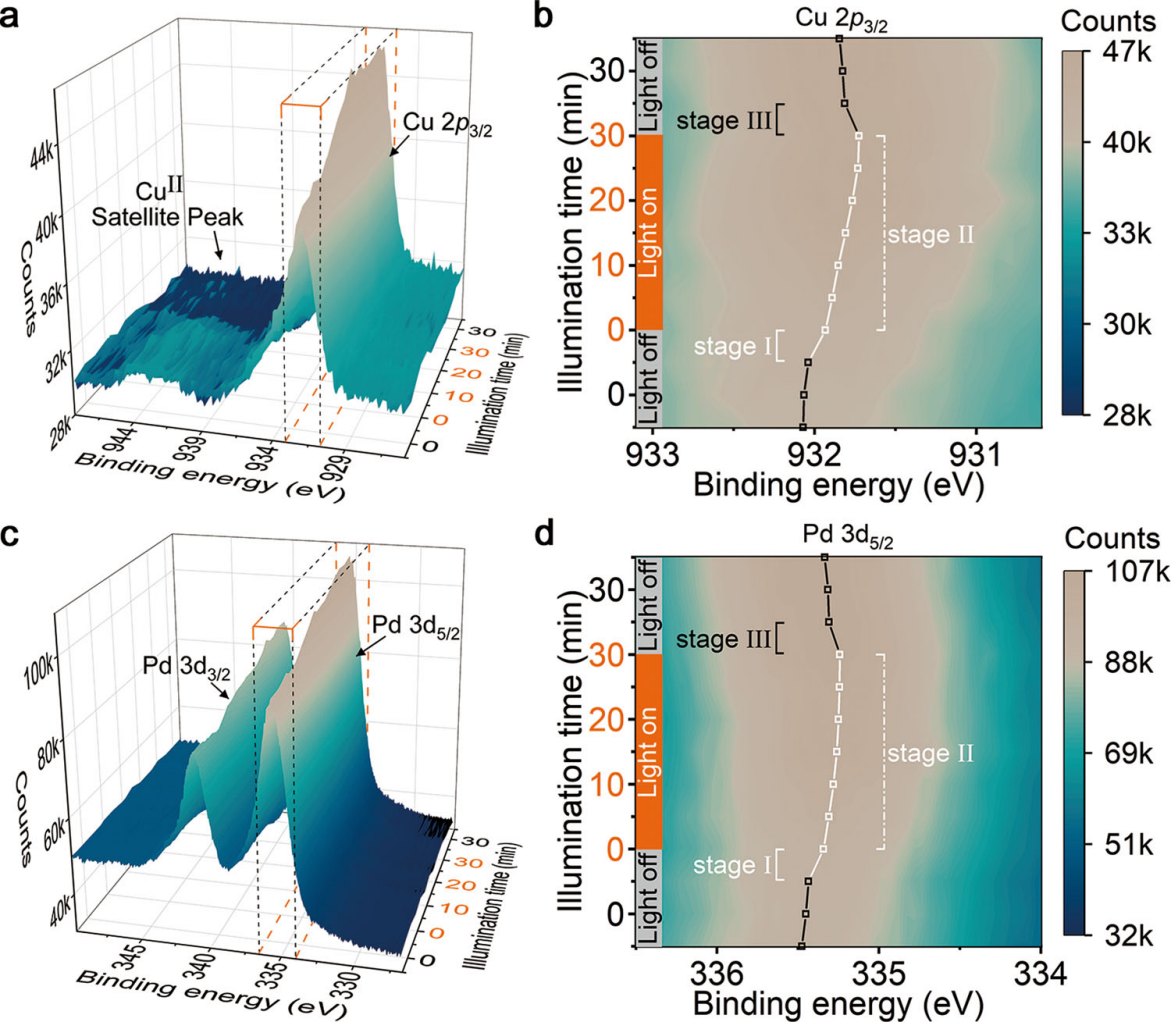
Deciphering plasmon-induced electron behavior via in situ NAP-XPS characterization. **a** and **b** In situ NAP-XPS spectra of Cu 2*p*_3/2_ (**a**) and contour plot for the amplified top view of the selected box-shaped area (**b**). **c**, **d** In situ NAP-XPS spectra of Pd 3*d*_5/2_ (**c**) and contour plot for the amplified top view of the selected box-shaped area (**d**). The in situ NAP-XPS data are recorded continuously for 1 h over Au rod@CuPd_2_ at 0.25 mbar CO_2_ atmosphere and light illumination is introduced into the catalytic system in the middle 30 min (marked in orange color). Representative peak positions are marked with open squares in (**b**) and (**d**). The squares and lines in black and white indicate unilluminated and illuminated conditions, respectively.

The direct charge transfer mechanism provides a reasonable explanation for the unique selectivity in plasmon-induced catalytic process^30–32^. Nevertheless, in the case of our work, a critical issue is that the energy of a single low-energy photon (~1.55 eV for 800 nm light) is insufficient for reaching the energy barrier of CO_2_RR without adding sacrificial agent. To gain insight into such a low-energy photon utilization ability for CO_2_RR, we employ in situ NAP-XPS to examine the plasmon-induced light-matter interaction in the catalytic system. All NAP-XPS spectra have been calibrated with the Au 4 *f* core level of Au foil, which is mounted beside the sample (see Fig. S20). Under the dark condition, no obvious fluctuation arises in the binding energy of Cu (Fig. 2a, b). Once turning on the light, the binding energy of the Cu peak instantaneously decreases (Stage I), and then is gradually reduced (Stage II) under subsequent light illumination. When the light is turned off, the binding energy of Cu increases immediately (Stage III) but cannot return to the initial level. Based on these observations, we can infer that the changes in Cu involve two conceivable processes, where one is fast and reversible (Stage I and III), and the other is slow and irreversible (Stage II). For Stage I and III, electron energy fluctuation appears when the interaction of incident light with catalyst changes. This phenomenon can be attributed to the accumulation of hot electrons above the Fermi level (*E*_f_), whose lifetime typically lasts only a few hundred femtoseconds^24,33^. For Stage II, it is accompanied by the gradual disappearance of the Cu^II^ satellite peak in Fig. 2a, suggesting that the Cu elements are gradually reduced under light illumination by the hot electrons generated by LSPR relaxation (see also Figs. S21 and S22). Such a chemical state change is relatively sluggish and can be responsible for the slow process. By analyzing the changes of Pd 3*d* NAP-XPS spectra in Fig. 2c, d, we can confirm that Pd shows similar three-stage behavior to Cu 2*p*_3/2_. From the viewpoint of catalysis, the slow photoreduction and the fast hot-electron accumulation above the *E*_f_ are both beneficial to improve the chemical potential of the catalyst^34,35^, which is vital to break its catalytic limitation. In the meantime, we also monitor the C 1 *s* spectra through in situ characterization. As shown in Fig. S23, two peaks at 284.4 and 293.2 eV attributed to the adsorbed C species (C_ad_ species) and gaseous CO_2_ (g-CO_2_), respectively, can be observed. The relative increase in peak area (see Fig. S21) and positive shift of the binding energy of g-CO_2_ peak under light conditions represent the continuous adsorption of CO_2_ molecules and their conversion process to hydrocarbons^36–38^.

**Fig. 3:**
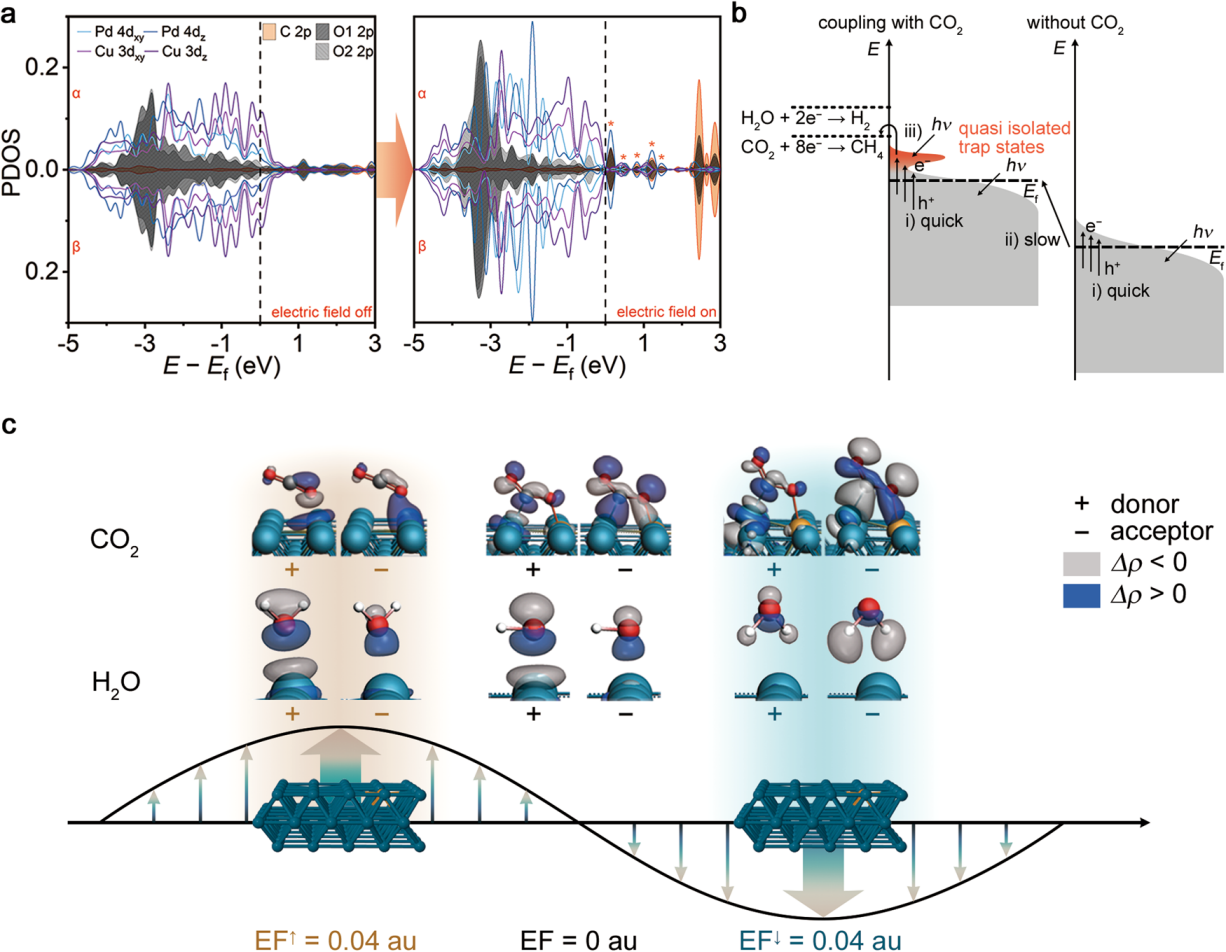
Theoretical calculations: the key role of local field in multiphoton absorption and energy transfer processes. **a** The projected density of states (PDOS) of CO_2_ adsorbed on a CuPd (100) surface in the absence (left) and presence (right) of electric field pointing toward the surface. The main quasi isolated trap states are marked with orange asterisks. **b** Schematic illustration for the plasmon-induced energy transfer in CO_2_RR, where the *y*-axis is energy (*E*) and the gray shading indicates filled electronic states. Three key processes for initiating and enhancing the plasmon-induced CO_2_RR include (i) fast excitation and accumulation of electrons above the Fermi level (*E*_f_), (ii) plasmon-induced slow photoreduction of Cu and Pd, and (iii) formation of new isolated trap state (origin shading) by coupling CO_2_ molecule with metal orbitals and re-excitation of the electrons above the Fermi level, which eventually result in the CO_2_RR process. **c** NOCV orbitals relevant to the most important deformation density in CO_2_ and H_2_O on a CuPd (100) surface in the absence (middle column) and presence of electric field toward (right column) or away from (left column) the surface. Donor NOCV on the left and acceptor NOCV on the right are displayed in each panel. Isovalue is set to 0.015.

To reveal the mechanism how CO_2_ is activated with the catalyst upon light irradiation, we further investigate the key role of unoccupied adsorbate states, by analyzing the projected density of states (PDOS, Fig. 3a) of our catalytic system consisting of catalyst and CO_2_ molecule. With CuPd as catalytic sites, the CO_2_ molecule can form hybrid states with the catalyst upon adsorption. Upon introduction of the electric field to the hybrid states, the calculation indicates that the state peaks of O1 2*p* and O2 2*p* shift toward lower energy and form a larger overlap area with the more localized Pd 4*d*_z_ and Cu 3*d*_z_ (parallel to the electric field) states, enhancing the stability of the key adsorption configuration^39^. More importantly, a series of new quasi isolated trap states emerge above the *E*_f_ in the presence of the electric field as illustrated in Fig. 3a. Compared to the continuous states near *E*_f_ of PdCu metal, these quasi isolated trap states can effectively extend the lifetime of hot electrons and increase the probability of electron re-excitation by another low-energy photon^40,41^, which makes the utilization of low-energy photons possible. Such a multiphoton absorption behavior can be corroborated by the superlinear law dependence between CH_4_ production rate and light intensity (see Figs. S24–S27)^19,20,22^. Taken together, the information gleaned by NAP-XPS and theoretical calculation can put forward the mechanism for plasmon-induced charge transfer in CO_2_RR as shown in Fig. 3b. Generally, there are three key processes for initiating and enhancing the plasmon-induced CO_2_RR, including (i) fast excitation for accumulating hot electrons above the Fermi level (*E*_f_), (ii) slow photoreduction process for elevating the chemical potential of the catalytic sites, and (iii) emergence of new quasi isolated trap states for re-exciting the electrons, gaining sufficient energy for triggering CO_2_RR. During the light-driven CO_2_RR, the three distinct processes work together to realize the utilization of low-energy NIR light for the reaction.

Given the key role of hybrid states in the mechanism, we further look into the hybrid orbitals composed of catalytic sites and CO_2_ or H_2_O molecules through DFT calculations. The optimized geometries and adsorption energies of molecules on CuPd (100) surface in different field conditions are illustrated in Fig. S28. Although the electron transfers from CuPd (100) surface to H_2_O and CO_2_ molecules exhibit similar trends (see Fig. S29), the CO_2_ and H_2_O display different natural orbitals for chemical valence (NOCVs) (Fig. 3c). In detail, when electric field points toward the metal surface, the electrons of CO_2_ are redistributed from O lone-pair orbital to its antibonding π* orbital, weakening the C–O bond and increasing the bond length. When the electric field points away from the metal surface, the lone-pair electrons transfer to the σ orbital between O and the metal atom. However, the electron transfer in adsorbed H_2_O molecules only oscillates between the bonding orbital of H_2_O and the orbital of Pd atoms. Thus the electric field provides scant assistance to the cleavage of the O–H bond. Based on these results, it is confirmed that the local electric field can facilitate the directional electron transfer to CO_2_ molecules, which contributes to the selective production of hydrocarbons.

### Performance enhancement based on catalytic mechanism

**Fig. 4:**
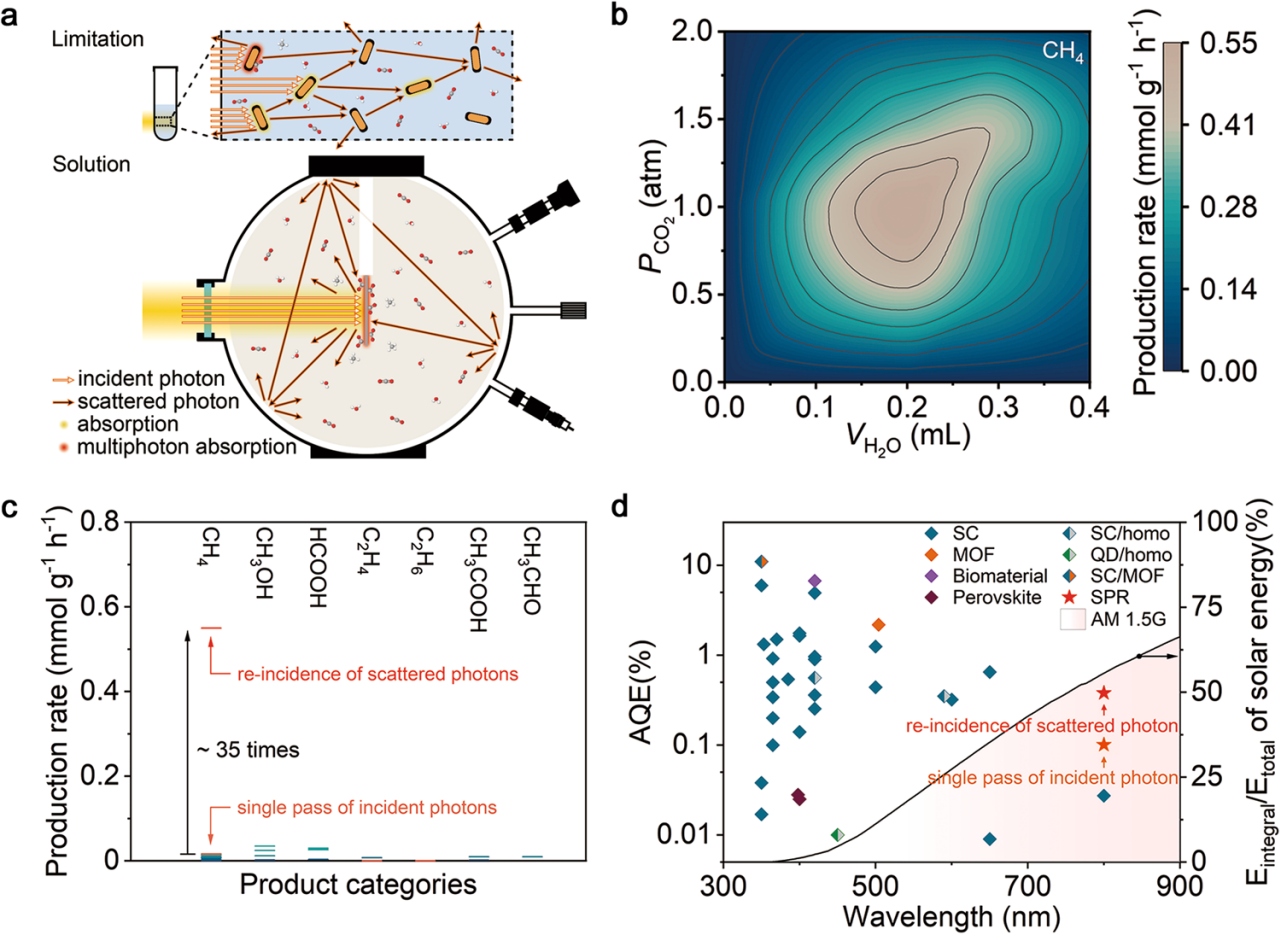
Optimized plasmonic catalysis: efficient utilization of scattered photons. **a** Schematic illustration of the gas-solid reaction, where mixed vapor of CO_2_ and H_2_O with centrally installed catalyst nanoparticles contained in a spherical reactor is illuminated with full-spectrum light. During the reaction, the scattered photons can be reflected to the catalyst surface, leading to efficient multi-absorption processes. **b** Average production rates of CH_4_ through plasmon-induced CO_2_RR using the gas-solid reaction device over Au rod@CuPd_2_ under different CO_2_ pressure and H_2_O volume. The catalytic experiments are carried out in the mixed vapor of CO_2_ and H_2_O without any sacrificial agent under 400 mW cm^–2^ full-spectrum light illumination. **c**, **d** The comparison of production rates (**c**, see Table S5) and AQEs (**d**, see Table S6) of Au rod@CuPd_2_ reported in this work with the records reported in the literature for the last 5 years on artificial photosynthesis, which directly convert CO_2_, H_2_O and sunlight into compounds without sacrificial agent.

Upon demonstrating the opportunity of utilizing low-energy photons in plasmon-induced CO_2_RR, it remains an open question whether it is possible to further improve the photon utilization for enhanced multiphoton absorption. Given the super-linear relationship between plasmonic catalytic rate and photon flux (i.e., the light intensity, Fig. S26), the inevitable scattering loss of incident light becomes the main limitation of the conventional reaction system (see Fig. 4a). To overcome the limitation, we design a gas-solid reaction system with a spherical structure to realize the re-incidence of scattered photons, which works only for the plasmonic catalytic system rather than conventional photocatalysis. By optimizing the volume of H_2_O and the partial pressure of CO_2_, the CH_4_ production rate of the light-driven CO_2_RR performed in such a system reaches 0.55 mmol g^−1^ h^−1^ (7.5 mmol g_Cu_^−1^ h^−1^) under 400 mW cm^−2^ full-spectrum light illumination (Fig. 4b), ca. 35 times higher than that in the reaction system allowing only a single pass of the incident photon. To verify the catalytic nature, turnover number (TON) is calculated (see Supplementary Text for detail). A TON of 9.6 per exposed Cu atoms is obtained after 3 h of illumination given that no catalyst deactivation has been observed in long-term operation. In the meantime, the control experiments are also performed in the dark at an elevated temperature (see Fig. S30 and Table S5), which matches the local temperature on the surface of catalyst nanoparticles under photoexcitation, to confirm the indispensability of light illumination. This conversion rate, enabled by the reaction system design taking advantage of plasmonic catalysis mechanism, far exceeds the current literature record (Fig. 4c and Table S6). Notably, the AQE in the gas-solid reaction at 800 nm is promoted to 0.38%. Such a superior photoconversion efficiency under low-energy NIR light illumination, which is even comparable with the efficiencies of the representative works in CO_2_RR with high-energy photons, allows our Au rod@CuPd_2_ catalyst to hit the record-high CO_2_RR AQEs under NIR light illumination (Fig. 4d and Table S7).

## Discussion

This work emphasizes the irreplaceable role of plasmon-induce local fields in the solar-to-chemical energy transfer process, providing a riveting picture of the potential of plasmon-induced catalysis toward achieving broadband artificial photosynthesis, particularly with the utilization of low-energy photons, and renders new insights for pushing forward the future development of plasmonic catalysis. Such a technique for low-energy photon utilization can potentially be integrated with the existing techniques available for UV and visible light in the future.

## Methods

### Materials

Potassium(II) tetrachloropalladate (K_2_PdCl_4_, 99.99%) was obtained from Beijing HWRK Chem Co., LTD. Sodium borohydride (NaBH_4_, 98%) was obtained from Sigma Aldrich. ^13^CO_2_ isotope was obtained from Sigma Aldrich. Copper(II) chloride dihydrate (CuCl_2_·2H_2_O) was obtained from Aladdin. Gold(III) chloride tetrahydrate (HAuCl_4_·4H_2_O, 99.95%), ascorbic acid (AA, 99.7%), hexadecyl trimethyl ammonium bromide (CTAB, 99.0%) and hexadecyl trimethyl ammonium chloride (CTAC, 98.0%) were purchased from Sinopharm Chemical Reagent Co., Ltd. The water used in all experiments was deionized. All the chemical reagents were used as received without further purification.

### Synthesis of Au rods

High-quality Au rods encircled by {100} and {110} facets were prepared through a modified seed-mediated growth process^42^. In the preparation of seed solution, an aqueous solution of CTAB (0.2 M, 2.25 mL) was mixed with 2.25 mL of 0.5 mM HAuCl_4_, and kept stirring in a 20 mL glass vessel. Subsequently, 157.5 μL ice-cold aqueous solution of NaBH_4_ (16.4 mM) was quickly added. Then the seed solution was kept vigorous stirring, resulting in the formation of a brownish yellow solution. After stirring for 1 min, it was kept at 30 °C for 30 min before further use.

In a typical synthetic process of Au rods, 195 mL CTAB (0.1 M) was kept in a 250 mL conical flask under magnetic stirring at room temperature. Subsequently, 5 mL aqueous solution of HAuCl_4_ (20 mM) and 5 mL aqueous solution of AgNO_3_ (4 mM) were injected into the conical flask successively. Then the aqueous solutions of AA (0.1 M) were dropped into the above solution until the color of the solution turned from orange to colorless. After another 300 μL AA (0.1 M) was added, 1 mL prepared seed solution was quickly added into the flask with a pipette. After reaction for 1 h, the products were centrifuged with deionized water for three times at 11,000 *rpm* for 10 min. The precipitation was dispersed in deionized water and measured by inductively coupled plasma mass spectrometry (ICP-MS) for further use.

### Synthesis of Au rod@CuPd_2_

The co-deposition of Cu and Pd atoms on Au rod was achieved via an epitaxial growth method. In a typical synthetic process for Au rod@CuPd_2_ sample, 70.8 mL aqueous solution containing 0.1 M hexadecyl trimethyl ammonium chloride (CTAC) was kept in a 100 mL round-bottom flask under magnetic stirring at 50 °C, followed by the addition of a suspension containing 4 mg Au rods. Then the aqueous solutions of K_2_PdCl_4_ (1 mL, 10 mM) and CuCl_2_ (1 mL, 10 mM) were added into the flask. Subsequently, 2.4 mL aqueous solution of ascorbic acid (0.1 M) was added into the flask with a pipette immediately. After reaction for 1 h, the products were centrifuged with deionized water for three times at 9500 *rpm* for 10 min before being re-dispersed in 4 mL deionized water.

### Synthesis of Au rod@CuPd_*x*_-*y*

Other Au rod@CuPd_*x*_-*y* samples were synthesized by following similar procedures except for the use of different amounts of K_2_PdCl_4_ and CuCl_2_. The usage of K_2_PdCl_4_ and CuCl_2_: (1) 1.2 mL K_2_PdCl_4_ and 800 μL CuCl_2_ for Au rod@CuPd_2.5_, (2) 1.4 mL K_2_PdCl_4_ and 600 μL CuCl_2_ for Au rod@CuPd_3.6_, (3) 1.6 mL K_2_PdCl_4_ and 400 μL CuCl_2_ for Au rod@CuPd_6.1_, (4) 1.8 mL K_2_PdCl_4_ and 200 μL CuCl_2_ for Au rod@CuPd_8.8_, (5) 1.9 mL K_2_PdCl_4_ and 100 μL CuCl_2_ for Au rod@CuPd_14.2_, (6) 200 μL K_2_PdCl_4_ and 200 μL CuCl_2_ for Au rod@CuPd_2_-0.1, (7) 500 μL K_2_PdCl_4_ and 500 μL CuCl_2_ for Au rod@CuPd_2_-0.2, (8) 1.5 mL K_2_PdCl_4_ and 1.5 mL CuCl_2_ for Au rod@CuPd_2_-0.9, and (9) 2.0 mL K_2_PdCl_4_ and 2.0 mL CuCl_2_ for Au rod@CuPd_2_-1.2.

### In situ NAP-XPS characterization

In situ NAP-XPS was performed at the X-ray Photoelectron Spectroscopy and UV Photoelectron Spectroscopy Endstation (BL24A1) in the National Synchrotron Radiation Center (NSRRC). The apparatus was composed of three chambers, including a sample load-lock chamber, a preparation chamber and an analysis chamber. The analysis chamber was equipped with an SPEC Phoibos 150 energy analyzer. The catalyst was dropcast onto a clear silicon wafer, and dried at room temperature. The samples were then loaded into the load-lock chamber and delivered to preparation chamber for 10 min Ar ion sputtering treatment. High-purity CO_2_ (99.9999%) was introduced into the analysis chamber with the partial pressure maintained at 0.25 mbar. Afterwards, the samples were delivered to the analysis chamber. NAP-XPS were recorded with 1170 eV photons while a Xe lamp source (PLS-SXE300D, Beijing Perfectlight) was used as a light source. The Cu 2*p*, Pd 3*d* and C 1*s* spectra were recorded continuously for 1 h and light illumination was introduced into the analysis chamber in the middle 30 min. The incident photon energy was calibrated against Au 4*f* core level measured with an Au foil mounted beside the sample.

### Sum frequency generation vibrational spectroscopy characterization

We performed the offline SFG-VS study on Au rod@CuPd_2_ with a home-built SFG-VS spectrometer. Briefly, an 1 kHz Ti:Sapphire laser amplification system (Spitfire Ace, Spectra-Physics) was employed to produce a ~80 fs pulse with the center wavelength at 800 nm and a pulse energy of 6 mJ. About 3 mJ of this output was fed into an optical parametric amplifier (TOPAS Prime, Light Conversion) to generate the femtosecond broadband infrared (IR) pulse, while another portion of the 800 nm fundamental beam (~1.2 mJ) was guided into a 4f pulse shaper to produce the narrowband upconversion pulse centered around 800 nm with a FWHM of ~7 cm^−1^. The broadband IR and narrowband upconversion pulses overlapped at the sample surface both spatially and temporally with their incident angles fixed at 45° and 63° against the surface normal, respectively. The sum frequency generation signals were acquired in a reflective geometry with a high-resolution optical spectrometer (SR-500i-A, Andor Inc.) coupled with a CCD detector (Du970P-BVF, Andor Inc.). Before the SFG-VS measurements, the sample films were deposited on the flat side of a CaF_2_ half cylinder support and washed with water vapor for 20 min to totally remove the hydrocarbon surfactant. The newly prepared films were transferred into CO_2_-saturated ultrapure water environment enclosed by a Teflon vessel, followed by the exposure to the broadband irradiation (Xe lamp) for 20 min. During the offline SFG-VS experiment, the averaged pulses of IR and upconversion pulses were kept at 3.0 and 9.0 μJ, respectively, for Au rod@CuPd_2_ at *ppp* (p-SFG, p-Vis, and p-IR) and *ssp* (s-SFG, s-Vis, and p-IR) polarization to avoid any surface damage. All SFG signals were averaged for 600 k shots.

### Characterization

Transmission electron microscopy (TEM) images were taken on a Hitachi Model H-7700 microscopy at an accelerating voltage of 100 kV. High-resolution transmission electron microscopy images and energy dispersive spectroscopy mapping profiles were recorded on a JEOL ARM-200F field-emission transmission electron microscope at an accelerating voltage of 200 kV. Powder XRD patterns were collected using a Japan Rigaku DMax-γA rotating anode X-ray diffractometer equipped with diffracted beam graphite monochromator (Cu Kα radiation (λ = 1.54178 Å)). The concentrations of various elements in the prepared samples were measured with a Thermo Scientific PlasmaQuad 3 inductively-coupled plasma mass spectrometry (ICP-MS) after dissolving them with a mixture of HCl and HNO_3_ (3:1, volume ratio). Ultraviolet-visible (UV-Vis) extinction spectra were recorded in the spectral region of 300–1000 nm with an Agilent Technologies Cary 60 spectrometer. The samples were diluted with deionized water to the same concentration before being measured in a quartz cuvette.

### Photocatalytic CO_2_ reduction reaction measurements

The light-driven catalytic CO_2_RR was performed in a homemade quartz tube. Typically, 1 mg catalyst sample (based on inductively coupled plasma mass spectrometry, ICP-MS measurements) was dispersed in deionized water (18.25 MΩ cm) in the tube to reach a total suspension volume of 4 mL, which was then vacuumed and saturated with high-purity CO_2_ (99.9999%) for three times and sealed with a glass stopper after filled with 1 atm high-purity CO_2_ (99.9999%). Subsequently the suspension was vigorously stirred, and illuminated using a Xe lamp source (PLS-SXE300D, Beijing PerfectLight) at a light intensity of 400 mW cm^–2^ (full-spectrum light). The resultant reaction gas was then analyzed by gas chromatography (GC, 7890 A, Agilent) equipped with a flame ionization detector (FID). The control experiments in dark environment were performed under the same conditions except the exclusion of light with an aluminum foil or the exclusion of carbon dioxide with high-purity Ar (99.9999%).

### Photocatalytic CO_2_ reduction measurements in a gas-solid biphase reactor

The light-driven catalytic CO_2_RR was performed in a 10 cm diameter spherical reactor equipped with a 1 cm diameter quartz window as the light inlet. The inside of the spherical reactor was uniformly coated with foamed polytetrafluoroethylene, whose reflectance is 0.978, 0.991, 0.989 and 0.988 at 400, 600, 800 and 1000 nm, respectively. Typically, 0.5 mg catalyst sample (based on ICP-MS measurements) was dispersed evenly on a 3 cm diameter quartz wool sheet, which was then gently dried in an inert atmosphere and installed in the center of the integrating sphere reactor facing the light inlet. Subsequently, 0–0.4 mL CO_2_-saturated water was dropped on the quartz wool sheet. After the reactor was sealed with the quartz window, high-purity CO_2_ (99.9999%) was introduced to purify the reactor and filled to 0–2 atm. Then a Xe lamp source (PLS-SXE300D, Beijing PerfectLight, 400 mW cm^–2^, full spectrum) was employed as a light source to irradiate the catalyst from the light inlet. The resultant reaction gas was then analyzed by gas chromatography (GC, 7890 A, Agilent) equipped with a FID. The control experiments in dark environment were performed under the same conditions except the exclusion of light with an aluminum foil or the exclusion of CO_2_ with high-purity Ar (99.9999%).

### Computational details

DFT calculations with periodic boundary conditions (PBC) were carried out using the program ADF-BAND 2019. Perdew-Burke-Ernzerhof exchange-correlation functional with DFT-D3 (BJ) dispersion correction and TZP basis set incorporated with ZORA approach to include the scalar relativistic effects were employed under the spin-unrestricted scheme. A 5 *×* *5 slab* with three atomic layers was used to mimic the Pd (100) surface with Cu doping. During the geometry optimizations, only the top layer was relaxed and the two bottom layers were frozen at the bulk positions. A *3* *×* *3* *×* *1 k*-point grid was used to sample the first Brillouin zone under the two-dimensional PBC. A $\varGamma$-only *k*-mesh was adopted in the energy decomposition analysis for extended systems (pEDA) with the extension of natural orbitals for chemical valence. Since the strong interaction of photons and plasmonic metal can result in an alternating electric field, which is localized near the metal surface following the law of sines (**E** = *A*sin*t*, where **E**, *A* and *t* represent electric field intensity, amplitude and time, respectively). In order to investigate the influence of the electric field induced by LSPR on the adsorbed molecules, the adsorption energy and bond length of these molecules were simulated under specific electric field conditions. Given the monotonicity and periodicity of the sine function, the three most representative conditions were studied. Specifically, when *t* = 0 or π, *t* = π/2 and *t* = 3π/2, and *A* = 0.04 au, the **E** values are 0, 0.04 and −0.04 au, respectively. Taken together, the external electric fields with the directions pointing toward/away the substrate were applied in simulations considering the field created from surface plasmon polariton.

## Supplementary information

Supplementary Information

## Data Availability

All data supporting the findings of this study are available in the article and its Supplementary Information. Source data for the following figures are provided with this paper. Fig 1b–d, Fig. 2a–d, Fig. 3a, Fig. 4b–d, Fig. S4a–g, Fig. S5a–g, Fig. S7, Fig. S9a and b, Fig. S10a and b, Fig. S11a and b, Fig. S13, Fig. S14, Fig. S15, Fig. S16a and b, Fig. S18a and b, Fig. S19a and b, Fig. S20, Fig. S21, Fig. S22a and b, Fig. S23a and b, Fig. S24a and b, Fig. S25, Fig. S26, Fig. S27. Source data are provided with this paper.

## Source data

Source Data
