# Publisher Correction: Near-infrared-featured broadband CO_2_ reduction with water to hydrocarbons by surface plasmon

**DOI:** 10.1038/s41467-023-37241-1

**Published:** 2023-03-22

**Authors:** Canyu Hu, Xing Chen, Jingxiang Low, Yaw-Wen Yang, Hao Li, Di Wu, Shuangming Chen, Jianbo Jin, He Li, Huanxin Ju, Chia-Hsin Wang, Zhou Lu, Ran Long, Li Song, Yujie Xiong

**Affiliations:** 1 Hefei National Research Center for Physical Sciences at the Microscale, School of Chemistry and Materials Science, and National Synchrotron Radiation Laboratory, University of Science and Technology of China, Hefei, 230026 Anhui China; 2 Institute of Energy, Hefei Comprehensive National Science Center, 350 Shushanhu Rd., Hefei, 230031 Anhui China; 3 Institute of Molecular Plus, Tianjin University, 92 Weijin Road, 300072 Tianjin, China; 4 National Synchrotron Radiation Research Center, Hsinchu, 30076 Taiwan; 5 Anhui Engineering Research Center of Carbon Neutrality, College of Chemistry and Materials Science, School of Physics and Electronic Information, and Key Laboratory of Functional Molecular Solids, Ministry of Education, Anhui Normal University, Wuhu, 241002 Anhui China

**Keywords:** Catalytic mechanisms, Photocatalysis, Characterization and analytical techniques, Photocatalysis, Artificial photosynthesis

Correction to: *Nature Communications* 10.1038/s41467-023-35860-2, published online 14 January 2023

In this article the affiliation details for Yujie Xiong were incorrectly given as ‘National Synchrotron Radiation Research Center, Hsinchu 30076, Taiwan’ but should have been ‘Anhui Engineering Research Center of Carbon Neutrality, College of Chemistry and Materials Science, School of Physics and Electronic Information, and Key Laboratory of Functional Molecular Solids, Ministry of Education, Anhui Normal University, Wuhu 241002 Anhui, China’. The original article has been corrected.

