# Supplementary material for: Near-infrared-featured broadband CO2 reduction with water to hydrocarbons by surface plasmon
Source: Nat Commun. 2023 Jan 14;14:221. doi: 10.1038/s41467-023-35860-2 (PMC9839746; doi:10.1038/s41467-023-35860-2)
Supplement: Supplementary file 1 — Supplementary Information [file 41467_2023_35860_MOESM1_ESM.pdf]

Supplementary Materials for

Near-infrared-featured broadband artificial photosynthesis for hydrocarbons by surface plasmon

Canyu Hu[1,2,6], Xing Chen[3,6], Jingxiang Low[1,6], Yaw-Wen Yang[4], Hao Li[5], Di Wu[1,2], Shuangming Chen[1], Jianbo Jin[1], He Li[1], Huanxin Ju[1], Chia-Hsin Wang[4], Zhou Lu[5], Ran Long[1]*, Li Song[1] and Yujie Xiong[1,2,5]*

[1]Hefei National Research Center for Physical Sciences at the Microscale, School of Chemistry and Materials Science, and National Synchrotron Radiation Laboratory, University of Science and Technology of China, Hefei, Anhui 230026, China.

[2]Institute of Energy, Hefei Comprehensive National Science Center, 350 Shushanhu Rd., Hefei, Anhui 230031, China.

[3]Institute of Molecular Plus, Tianjin University, 92 Weijin Road, Tianjin 300072, China.

[4]National Synchrotron Radiation Research Center, Hsinchu 30076, Taiwan.

[5]Anhui Engineering Research Center of Carbon Neutrality, College of Chemistry and Materials Science, School of Physics and Electronic Information, and Key Laboratory of Functional Molecular Solids, Ministry of Education, Anhui Normal University, Wuhu, Anhui 241002, China.

[6]These authors contributed equally.

Correspondence to: yjxiong@ustc.edu.cn (Yujie Xiong); longran@ustc.edu.cn (Ran Long)

This PDF file includes:

Supplementary Text
Figs. S1 to S30
Tables S1 to S7
References (1–57)

Supplementary Text

Determination of apparent quantum efficiency (AQE)

To obtain the AQE, the light was filtered by different monochromatic filters, and the AQE was calculated as following equation:

$$\mathrm{AQE} = \frac{8 \times mol_{\mathrm{CH_4}}}{P \times t \times S \times \frac{\lambda}{h\mathrm{c}}}$$

where $mol_{\mathrm{CH_4}}$, P, t, S, λ, h and c represent the molar number of generated CH_4, incident light intensity, irradiation time, irradiation area, incident wavelength, Planck constant, and speed of light, respectively.

Determination of turnover number (TON)

The volume of $CuPd_2$ shell:

$$V_{shell} = \pi\, r^2_{Au\,rod@CuPd_2} h_{Au\,rod@CuPd_2} - \pi\, r^2_{Au\,rod} h_{Au\,rod}$$

where r and h represent the section radius and length of nanorod, respectively.

The surface area of $CuPd_2$ shell:

$$S_{shell} = 2\pi r^2_{Au\,rod@CuPd_2} + 2\pi r_{Au\,rod@CuPd_2} h_{Au\,rod@CuPd_2}$$

The surface ratio of Cu atoms:

$$\mathrm{ratio}_{surface\,Cu} = \frac{2S_{shell}}{a^2_{CuPd_2}} / \frac{4V_{shell}}{V_{CuPd_2}}$$

where unit cell parameter $a_{\mathrm{CuPd_2}}$ = *ca.* 0.378 nm, and unit cell volume $V_{\mathrm{CuPd_2}}$ = *ca.* 0.054 nm^3 according to the Vegard's law.

The turnover number was calculated by the following equation:

$$\mathrm{TON}_{Cu\,exposed} = \frac{mol_{CO_2}}{\mathrm{ratio}_{surface\,Cu} \times mol_{total\,Cu}}$$

where mol_{CO_2} is the number of formed CH_4 molecules in 3 h of illumination. 3 hours were selected as a demonstration given that no catalyst deactivation was observed in long-term operation.

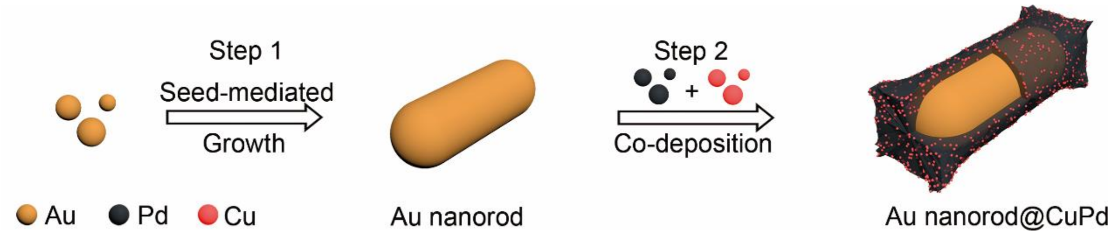

Fig. S1. Schematic illustration for the synthesis of Au rod@CuPd.

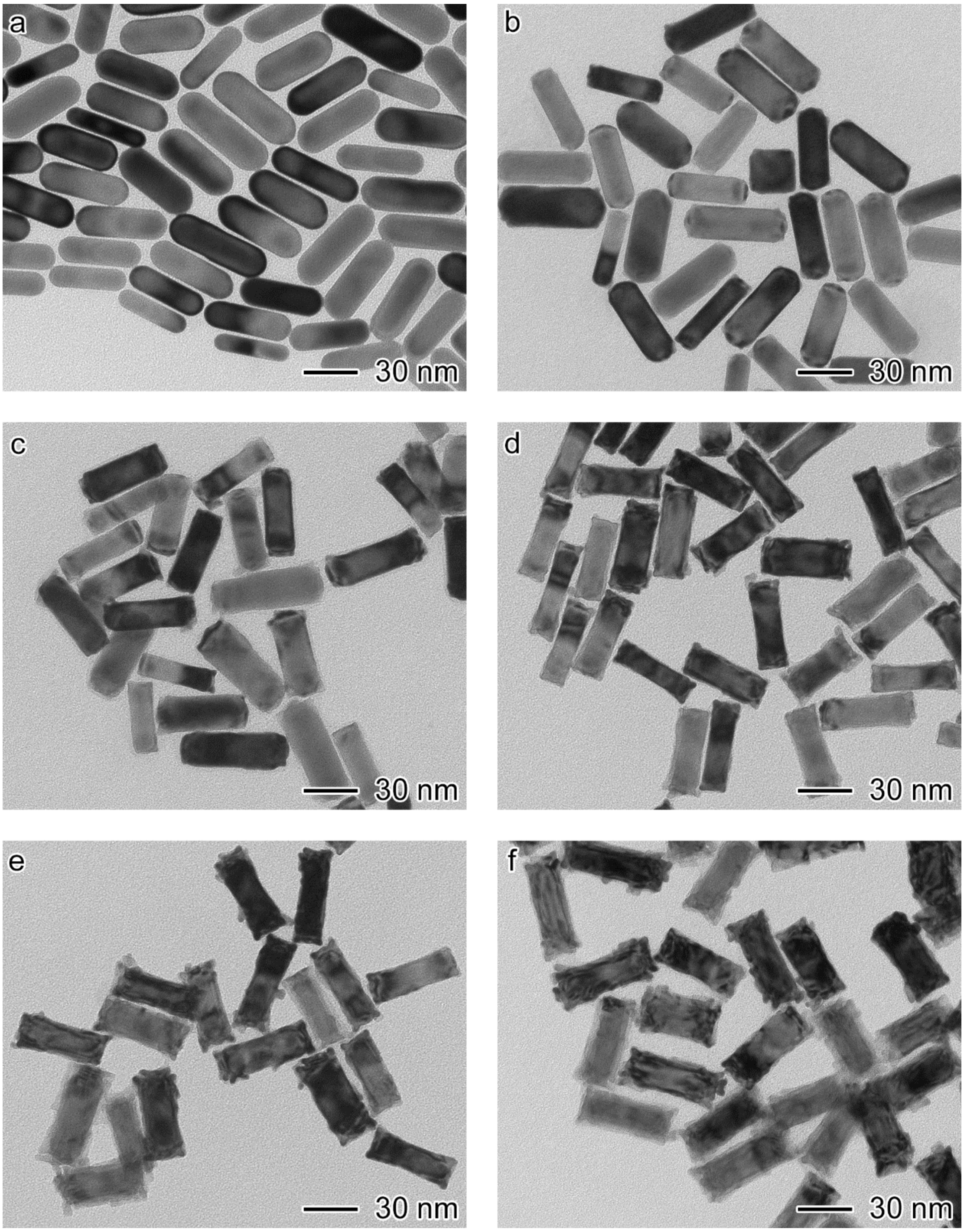

Fig. S2. (**a**–**f**) TEM images of Au rod (a), Au rod@$CuPd_2$-0.1 (b), Au rod@$CuPd_2$-0.2 (c), Au rod@$CuPd_2$-0.6 (d), Au rod@$CuPd_2$-0.9 (e), and Au rod@$CuPd_2$-1.2 (f).

As shown in Fig. S2a, the as-synthesized Au rods have an average diameter of 14 nm and length of 43 nm with an aspect ratio of 3.1.

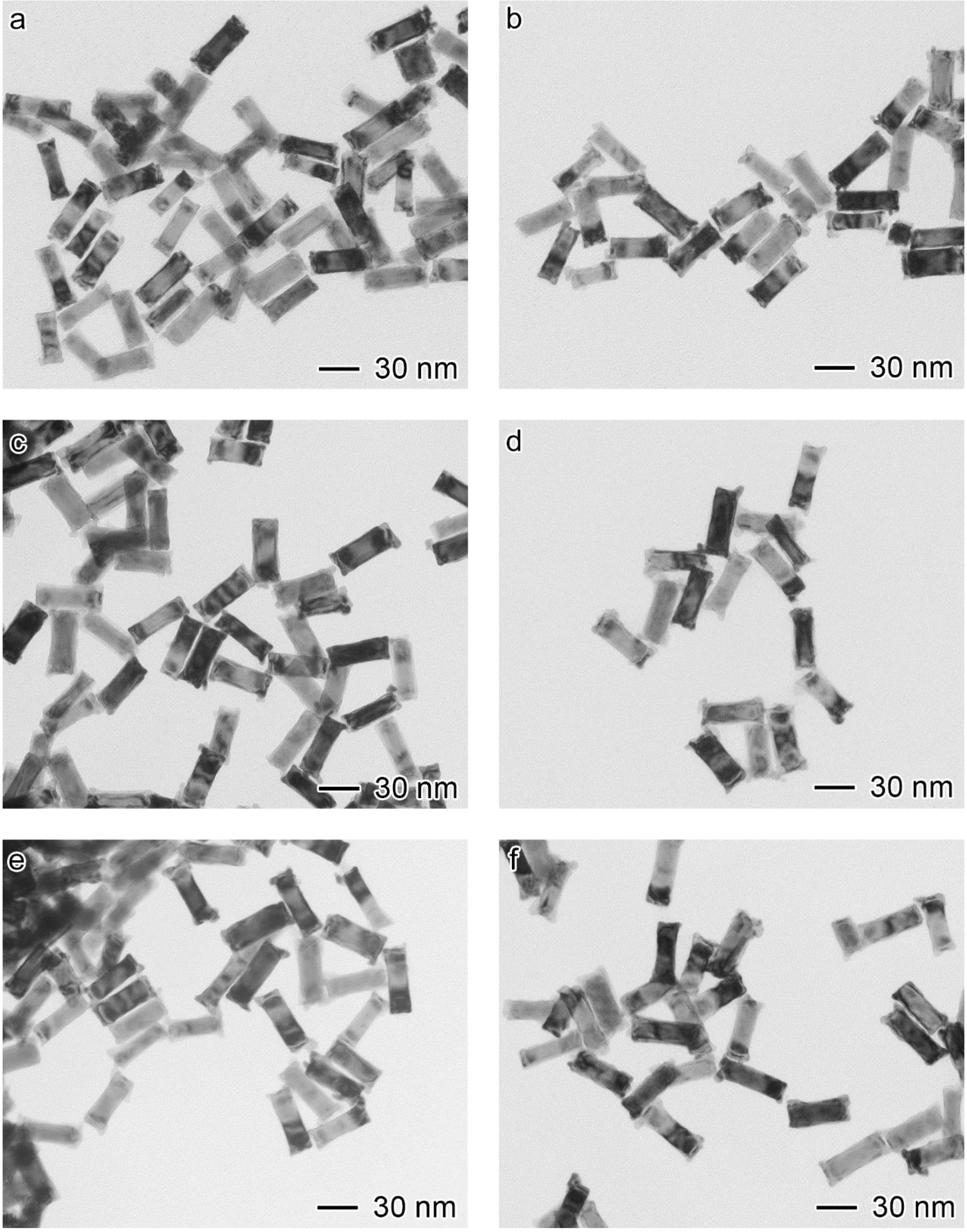

Fig. S3. (**a**–**f**) TEM images of Au rod@$CuPd_2$ (a), Au rod@$CuPd_{2.5}$ (b), Au rod@$CuPd_{3.6}$ (c), Au rod@$CuPd_{6.1}$ (d), Au rod@$CuPd_{8.8}$ (e) and Au rod@$CuPd_{14.2}$ (f).

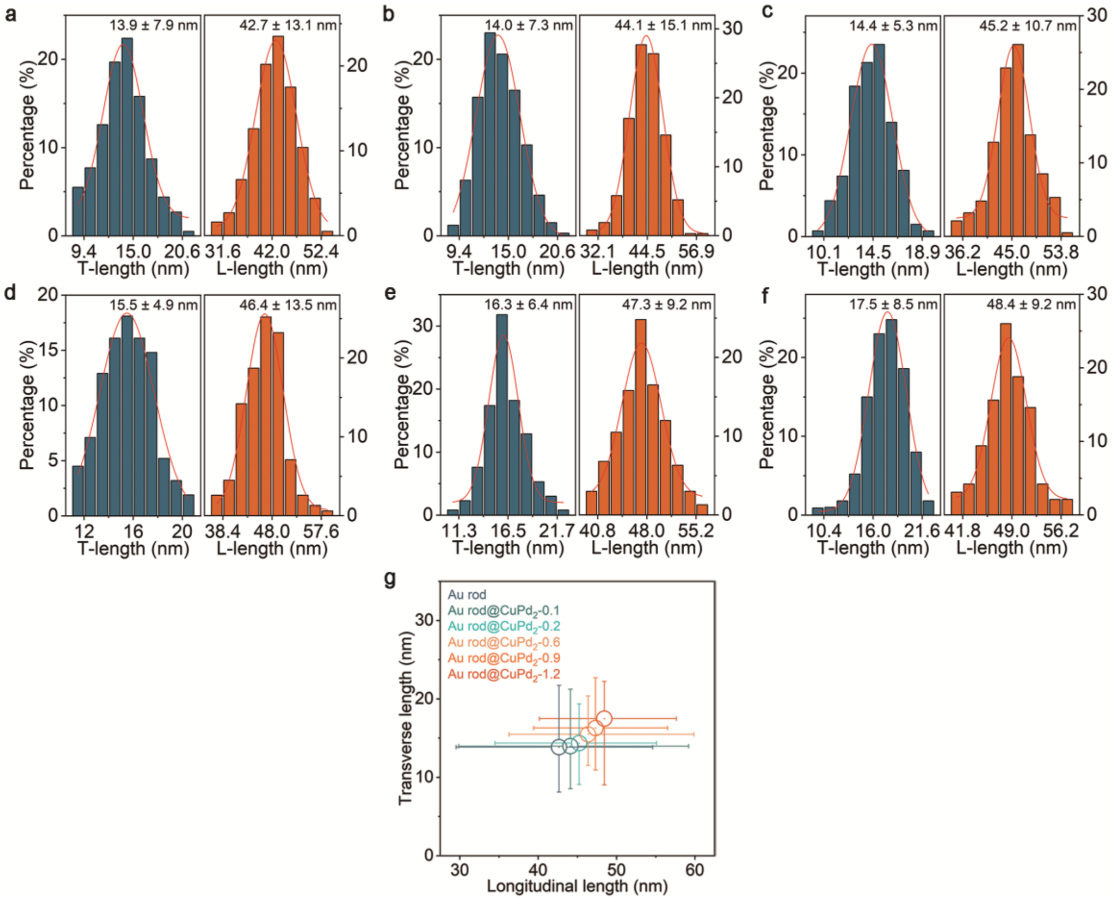

Fig. S4. (**a**–**f**) Transverse and longitudinal size distribution histograms for Au rods (a), Au rod@$CuPd_2$-0.1 (b), Au rod@$CuPd_2$-0.2 (c), Au rod@$CuPd_2$-0.6 (d), Au rod@$CuPd_2$-0.9 (e) and Au rod@$CuPd_2$-1.2 (f). (**g**) Size change trends in samples with different amounts of CuPd precursors. The error bars represent the standard deviation of the experiments.

Fig. S4 shows that the sample sizes become larger with the increasing amount of CuPd precursors. Especially, the increases in size preferentially emerge in the longitudinal length direction. This is because CuPd alloy favors depositing on both ends of the Au rods, which have a relatively high surface energy.

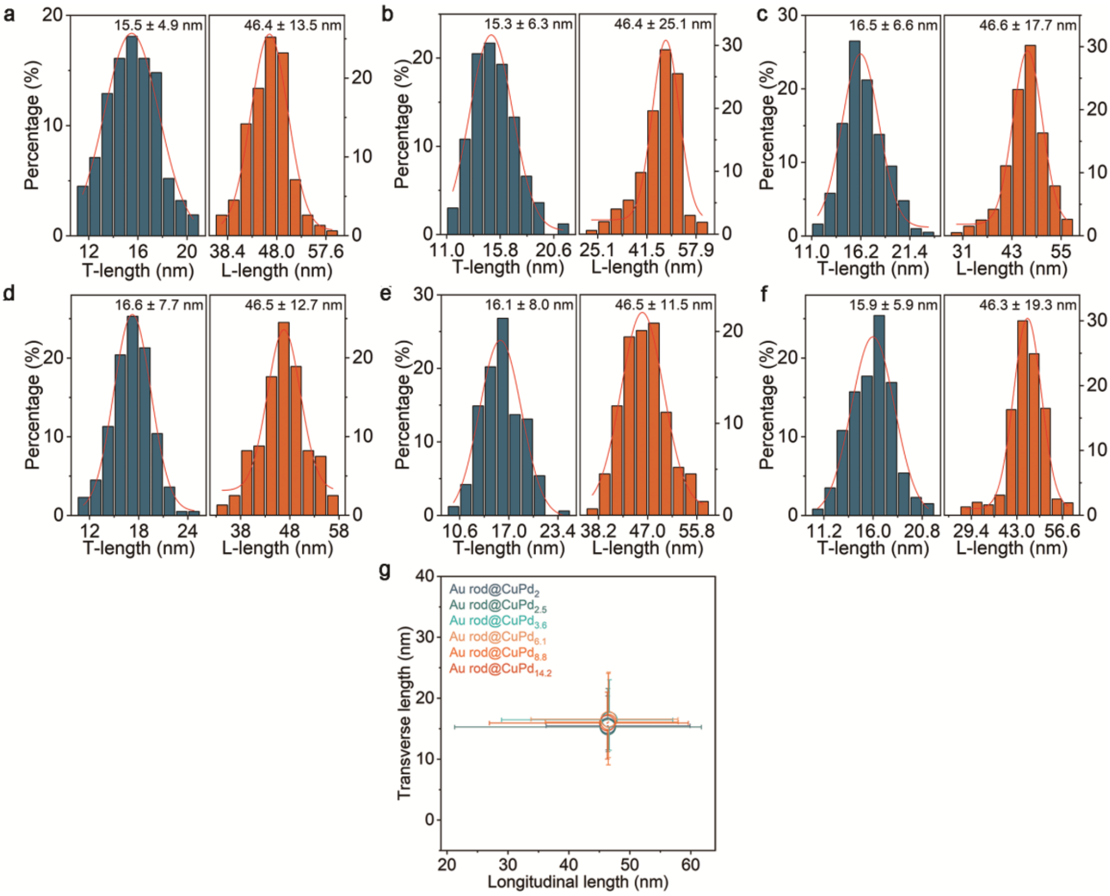

Fig. S5. (**a**–**f**) Transverse and longitudinal size distribution histograms for Au rod@$CuPd_2$ (a), Au rod@$CuPd_{2.5}$ (b), Au rod@$CuPd_{3.6}$ (c), Au rod@$CuPd_{6.1}$ (d), Au rod@$CuPd_{8.8}$ (e) and Au rod@$CuPd_{14.2}$ (f). (**g**) Size change trends in samples with different Cu/Pd ratios above. The error bars represent the standard deviation of the experiments.

Fig. S5 shows that the sample sizes have no obvious change when Cu/Pd ratios in the shell are altered.

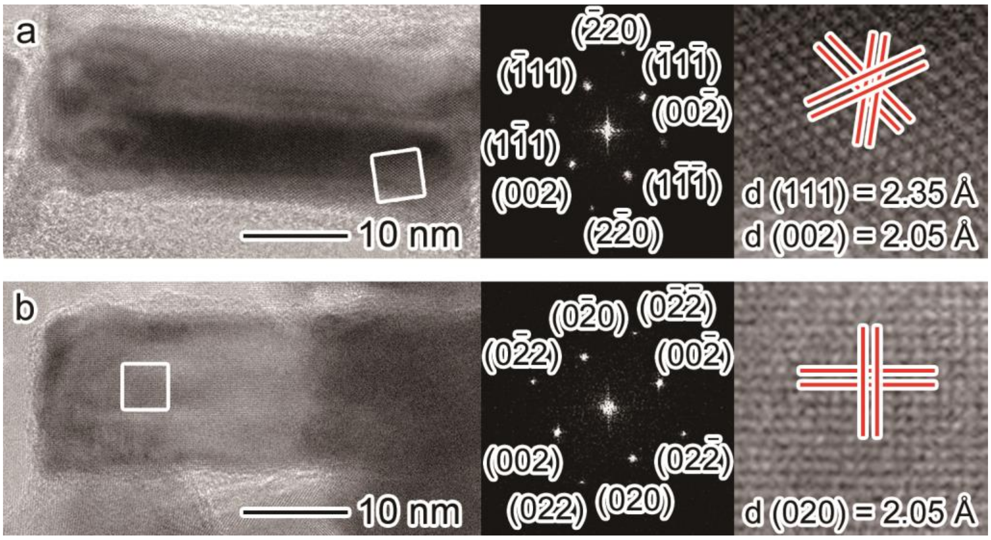

Fig. S6. (**a** and **b**) HRTEM image of Au rod@$CuPd_2$ recorded along [110] (a) and [100] (b) orientation with corresponding FFT pattern and magnified HRTEM image taken from selected area.

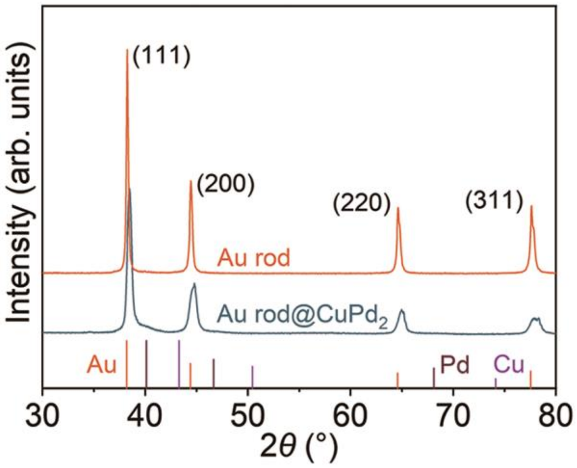

Fig. S7. Powder XRD patterns of as-prepared Au rods (orange line) and Au rod@$CuPd_2$ (cyan line).

XRD patterns (Fig. S7) further confirm the crystal structure of the prepared samples. Specifically, the XRD diffraction peaks of Au rod@$CuPd_2$ match well with the (111), (200), (220) and (311) planes of face-centered cubic Au rod, except for a minor shift and widening of the peaks under the influence of CuPd shell.

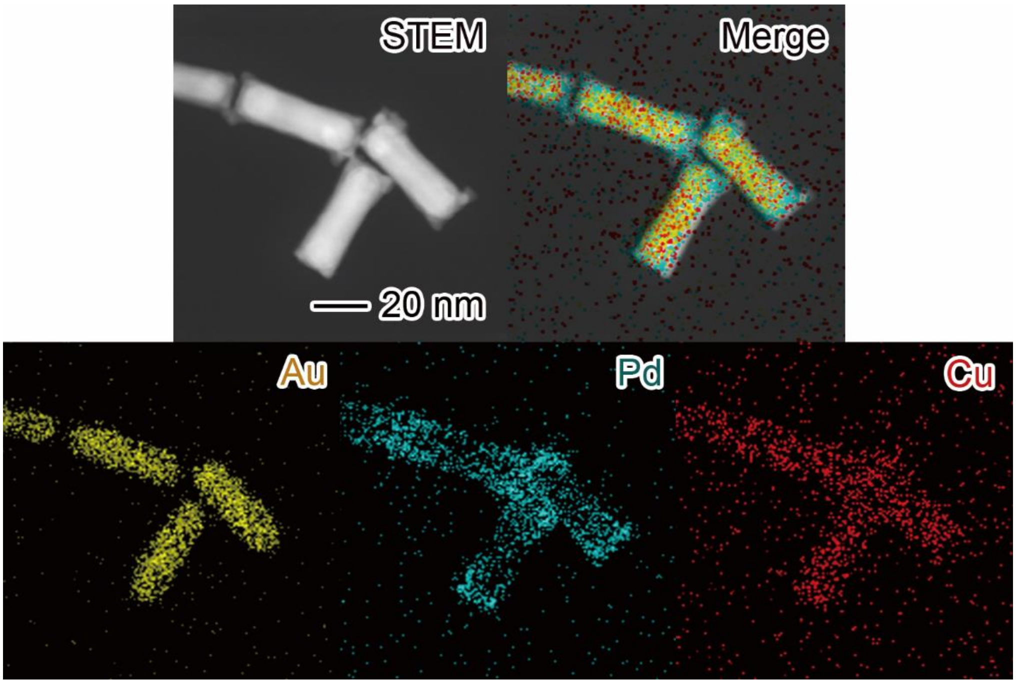

Fig. S8. Scanning transmission electron microscopy image for Au rod@$CuPd_2$ and corresponding EDS elemental mapping profiles for Au (orange), Pd (cyan), Cu (red) and mixed elements.

The elemental mapping profiles are shown in Fig. S8 and suggest that the Au element is concentrated in the core, while Pd and Cu elements are evenly distributed on the surrounding shell.

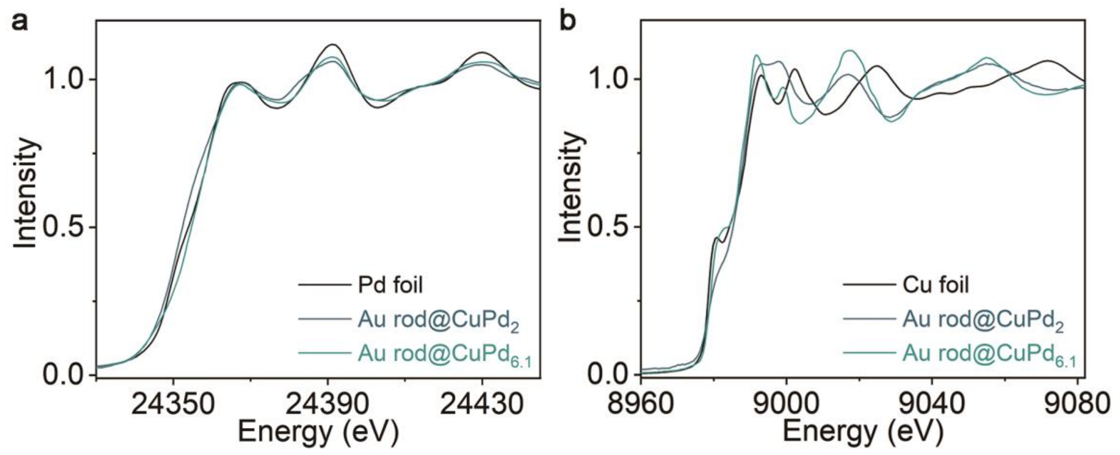

Fig. S9. (**a** and **b**) Normalized Pd K-edge (a) and Cu K-edge (b) XANES spectra of Au rod@$CuPd_2$ and Au rod@$CuPd_{6.1}$ in reference to Pd foil and Cu foil.

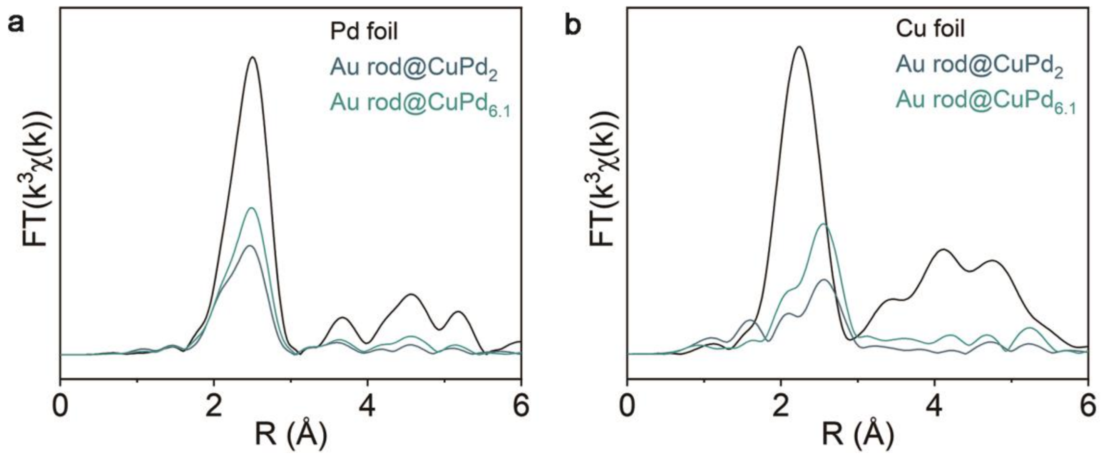

Fig. S10. EXAFS measurements. (**a** and **b**) k^3-weighted Fourier-transform Pd K-edge (a) and Cu K-edge (b) EXAFS spectra of Au rod@$CuPd_2$ and Au rod@$CuPd_{6.1}$ in reference to Pd foil and Cu foil.

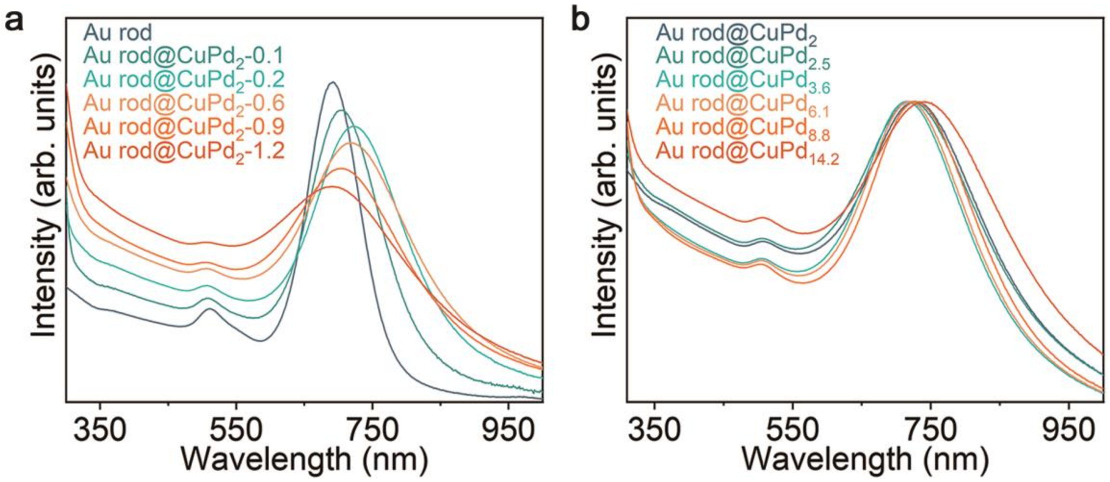

Fig. S11. Optical properties. (**a** and **b**) UV-vis extinction spectra of Au rod and Au rod@CuPd with different CuPd shell thicknesses (a) and different Cu/Pd molar ratios (b).

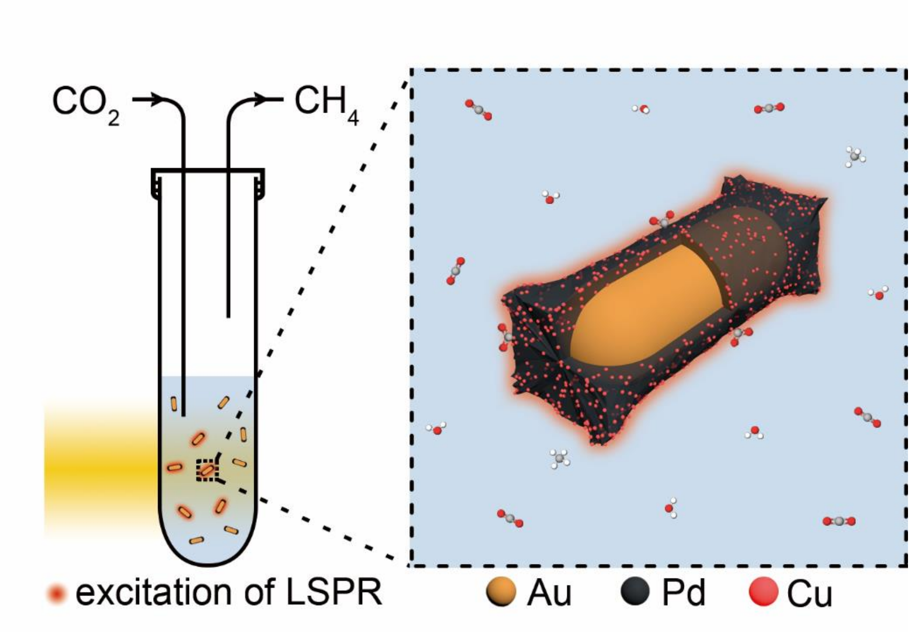

Fig. S12. Schematic illustration of the reaction where a CO_2-saturated solution containing Au rod@$CuPd_2$ nanoparticles in a quartz tube is illuminated with full-spectrum light (left) and the physiochemical processes involved in the reaction system where the nanoparticles scatter and/or absorb one or more photons for initiating CO_2RR (right).

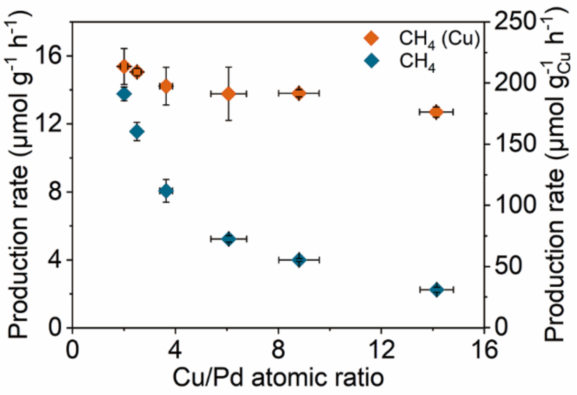

Fig. S13. Average production rates of CH_4 over Au rod@CuPd with different Cu/Pd atomic ratios (cyan dots) and those normalized by the amounts of Cu atoms (orange dots). The error bars represent the standard deviation of the experiments.

The catalytic experiments in Fig. S13 are carried out in CO_2-saturated water and CO_2 atmosphere without any sacrificial agent under 400 mW cm^{-2} full-spectrum light illumination.

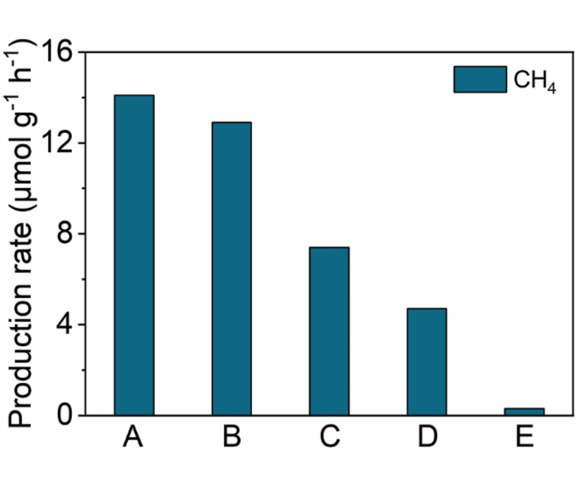

Fig. S14. Average production rates of CH_4 in light-driven CO_2RR in CO_2-saturated water for 3 h over Au rod@$CuPd_2$ under 400 mW cm^{-2} full-spectrum illumination (A), under simulated solar spectrum illumination (B), and under illumination with light filtered by a long-pass filter ($\lambda > 600$ nm, C), wideband-pass filter (600 nm $> \lambda >$ 450 nm, D) or UV cut filter ($\lambda < 450$ nm, E).

As shown in Fig. S14, thanks to the broad light extinction cross-section in the visible and near-infrared regions, the photocatalytic efficiency produced by low-energy light ($\lambda > 600$ nm) marvelously accounts for more than 60% of the total solar-to-chemical energy conversion efficiency.

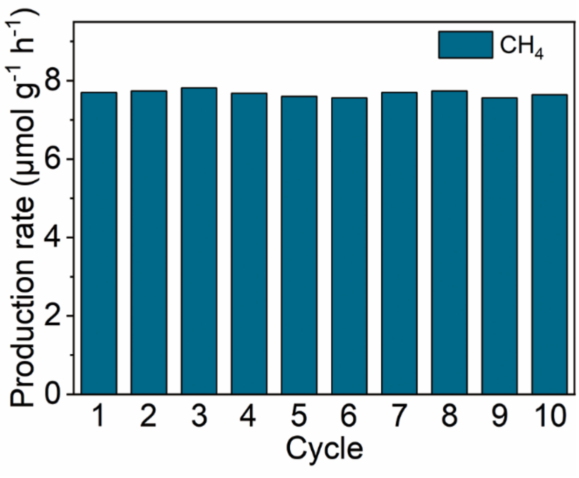

Fig. S15. Cycling tests (each run for 3 h) for Au rod@$CuPd_2$ under 800 nm light illumination.

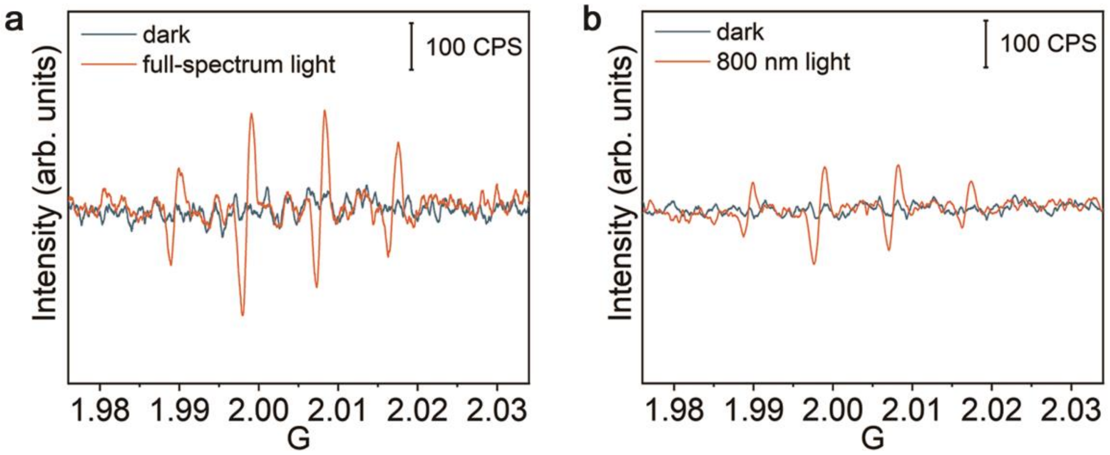

Fig. S16. (**a** and **b**) ESR spectra of DMPO in the presence of Au rod@$CuPd_2$ under (a) full-spectrum light and (b) 800 nm monochromatic light irradiation.

To verify the oxidized products formed during the reaction in CO_2-saturated water by Au rod@$CuPd_2$, we further employ electron spin resonance (ESR) spectroscopy to examine the system. With 5,5-dimethy-1-pyrroline N-oxide (DMPO) as a spin-trapping agent, a nearly 1:2:2:1 quartet signal is observed for nitroxide−OH spin adduct (DMPO-OH, $a^N = a^H = 1.49$ mT) under the full-spectrum irradiation in the presence of Au rod@$CuPd_2$ (Fig. S16a), confirming that the hydroxyl radical (•OH) is the main oxygen species produced from water oxidation by photogenerated holes. In addition, we also perform the characterization using the Xe lamp with 800 nm band-pass filter as a light source (Fig. S16b). A similar 1:2:2:1 quartet signal is obtained, suggesting that the low-energy photons can also drive CO_2RR and water oxidation using Au rod@$CuPd_2$ as a catalyst.

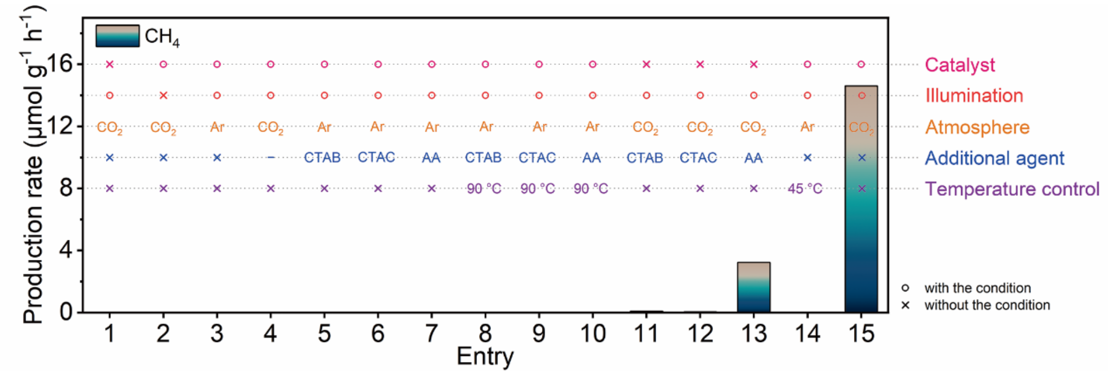

Fig. S17. CH_4 production rates of plasmon-induced CO_2RR over Au rod@$CuPd_2$ under various reaction conditions (see also Table S4). "–" stands for the case without H_2O.

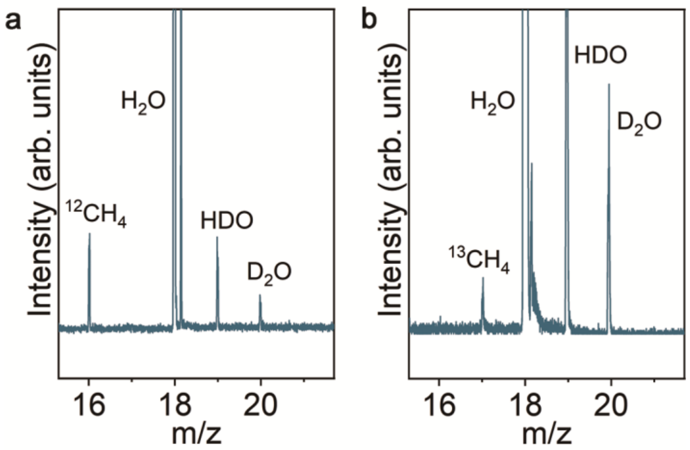

Fig. S18. (**a** and **b**) Mass spectra of gas products after CO_2RR using Au rod@CuPd$_2$ as a catalyst in $^{12}CO_2$ (a) and $^{13}CO_2$ (b) atmosphere. The reactions are conducted in CO_2-saturated water and 1 atm CO_2 atmosphere under 400 mW cm^{-2} full-spectrum illumination. The spectra are collected by an SVUV-PIMS.

In contrast to conventional mass spectrometry, the dissociation energy of SVUV-PIMS can be tuned by changing the light energy of synchrotron radiation, to avoid the dissociation of detected molecules during photoionization. Under this circumstance, the detected molecules exhibit unique mass-to-charge ratios. The *m*/*z* peaks at 18, 19 and 20 are attributed to H_2O, HDO and D_2O, respectively.

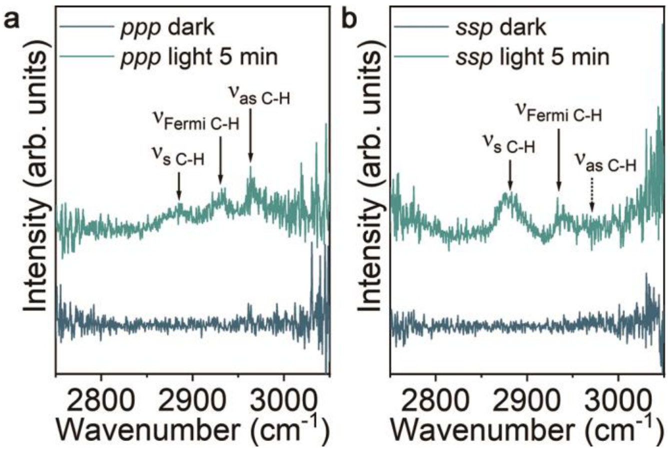

Fig. S19. (**a** and **b**) The offline SFG-VS spectra recorded during the light-driven CO_2RR over Au rod@$CuPd_2$. The dark blue lines are collected under the dark condition and the cyan lines are collected after illumination for 20 min in CO_2-saturated water. The data are collected with *ppp* (a) and *ssp* (b) polarization. The relative intensities of $\nu_{s\ C–H}$, $\nu_{Fermi\ C–H}$ and $\nu_{as\ C–H}$ for the *ppp* and *ssp* polarization are different, because of their difference in inherent sensitivity in the two different polarization modes.

The sum frequency generation is a second-order nonlinear process, which occurs when short infrared (~fs) and visible (~ps) laser pulses are overlapped in time and space. Due to interference effects, SFG-VS inherently excludes contributions from the bulk and typically only light from interfacial regions can be detected. As shown in Fig. S19, a and b, no SFG-VS signal exists before illumination.

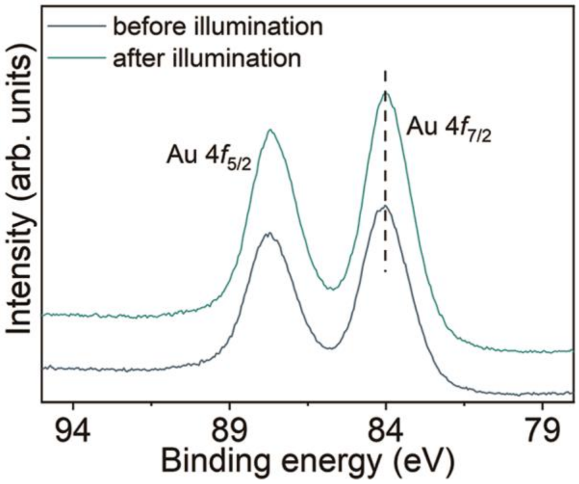

Fig. S20. Au 4f NAP-XPS spectra as reference Au foil before illumination (dark blue line) and after illumination (cyan line).

It is worth mentioning that comparing the Au 4*f* NAP-XPS spectra of Au foil as a reference before and after illumination, no obvious changes can be found, ensuring that the NAP-XPS spectra obtained in this work are not subjected to the possible energy drift of the synchrotron radiation source (Fig. S20).

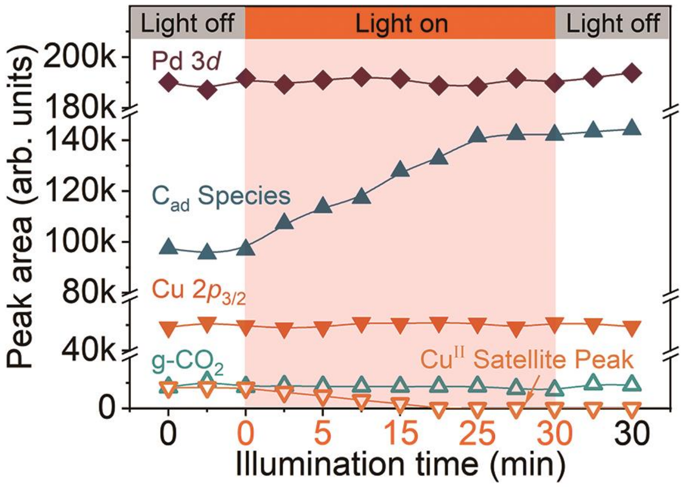

Fig. S21. Peak areas of Pd 3*d*, Cu 2*p* and C 1*s* NAP-XPS spectra under different illumination times.

In order to highlight the changes in the valence and content of elements, we investigate the area changes of different characteristic peaks of the Cu, Pd and C NAP-XPS spectra in Fig. 2a, c and Fig. S23, respectively. As shown in Fig. S21, the peak areas of Pd 3*d* and Cu 2*p* NAP-XPS spectra remain almost unchanged, indicating that the concentration or distribution of Pd and Cu in Au rod@$CuPd_2$ is kept stable before and after illumination. Notably, the Cu^{II} satellite peak gradually decreases and eventually disappears with the evolution of illumination time, illustrating that the Cu elements are gradually reduced under light illumination by the hot electrons generated by LSPR relaxation. As for g-CO_2, its peak area remains almost unchanged, implying that the pressure of CO_2 in NAP-XPS chamber is kept stable throughout the experiment.

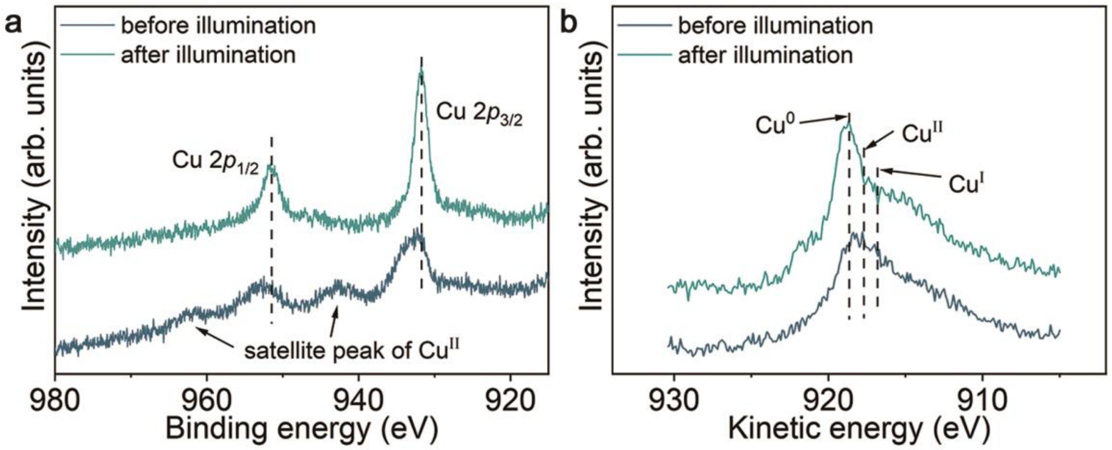

Fig. S22. (**a** and **b**) Cu 2*p* NAP-XPS spectra (a) and Cu LVV AES spectra (b) for Au rod@$CuPd_2$ before illumination (dark blue line) and after illumination for 30 min (cyan line).

The Cu 2*p* core-level spectra of Au rod@$CuPd_2$ are illustrated in Fig. S22a. Before illumination, Au rod@$CuPd_2$ shows a broad peak at 933.1 eV attributed to Cu^{II} $2p_{3/2}$ and a satellite peak at 942.8 eV, manifesting the presence of Cu^{II} species. After illumination for 30 min, the reduction of Cu^{II} $2p_{3/2}$ binding energy to 931.8 eV (for reference, the binding energy peaks of Cu^0 $2p_{3/2}$ and Cu^I $2p_{3/2}$ are located at 932.6 eV and 932.4 eV, respectively) and the disappearance of the Cu^{II} satellite peak clearly declare that Cu undergoes a photoreduction process, which is also corroborated by the shift of the Cu LVV Auger electron spectra to higher kinetic energy (see Fig. S22b) [2].

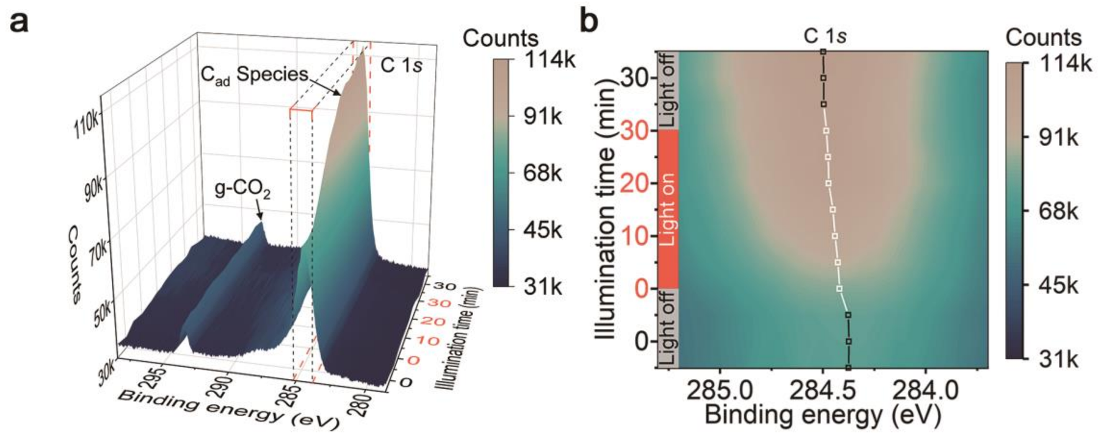

Fig. S23. (**a** and **b**) In situ NAP-XPS spectra of C 1*s* (a) and contour plot for the amplified top view of the selected box-shaped area (b). The in situ NAP-XPS data are recorded continuously for 1 h over Au rod@$CuPd_2$ at 0.25 mbar CO_2 atmosphere and light illumination is introduced into the catalytic system in the middle 30 min (marked in orange color). Representative peak positions are marked with open squares in b. The squares and lines in black and white indicate unilluminated and illuminated conditions, respectively.

We also pay attention to the evolution of C species adsorbed on the surface of Au rod@$CuPd_2$. Two peaks at 284.4 and 293.2 eV (see Fig. S23a) are attributed to the adsorbed C species (C_{ad} species) and gaseous CO_2 (g-CO_2), respectively. As revealed by summarized peak areas in Fig. S21, once the light is turned on, the peak area of C_{ad} species gradually increases to a stable value, suggesting that the surface plasmon can induce additional active sites for adsorption and activation of CO_2 molecules. As for g-CO_2, its peak area remains almost unchanged, implying that the pressure of CO_2 in NAP-XPS chamber is kept stable throughout the experiment. Moreover, the contour plot for the amplified top view of the selected box-shaped area (see Fig. S23b) clearly illustrates the shift of C 1*s* XPS peak position toward higher binding energy under continuous illumination. According to the previous literature, such a peak position shift can be attributed to the gradual replacement of sp^2-C species (binding energy around 284.0 eV) by sp^3-C species (binding energy around 284.8 eV), which is consistent with the hydrocarbon production by plasmon-induced CO_2RR.

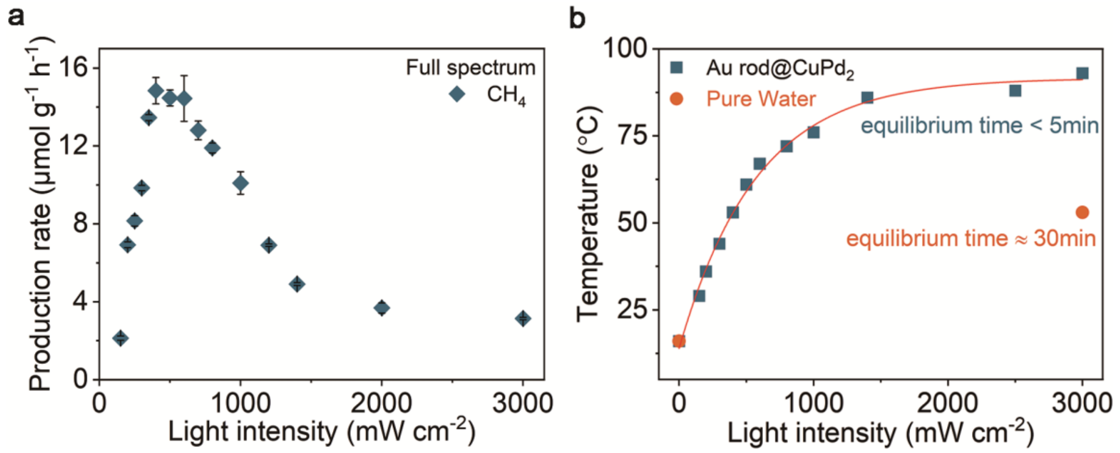

Fig. S24. (**a**) Average production rates of CH_4 as a function of light intensity (full-spectrum, CO_2-saturated water). (**b**) The changes in system temperature with the light intensity (full-spectrum, CO_2-saturated water). The error bars represent the standard deviation of the experiments.

To examine how photon energy is coupled into CO_2 activation, we perform CO_2RR by altering photon flux (i.e., light intensity) as the rate of charge-carrier formation in plasmonic metals. As shown in Fig. S24a, a volcano-shaped relationship emerges between the production rate of CH_4 and light intensity. This is because the photothermal effect of Au rod@$CuPd_2$ (see Fig. S24b) inevitably reduces the solubility of CO_2 in the reaction solution, resulting in a slowdown in CO_2RR, especially under intense light illumination. Therefore, for fundamental understanding, the relationship between reaction rate and solution temperature is investigated based on a series of temperature-control experiments.

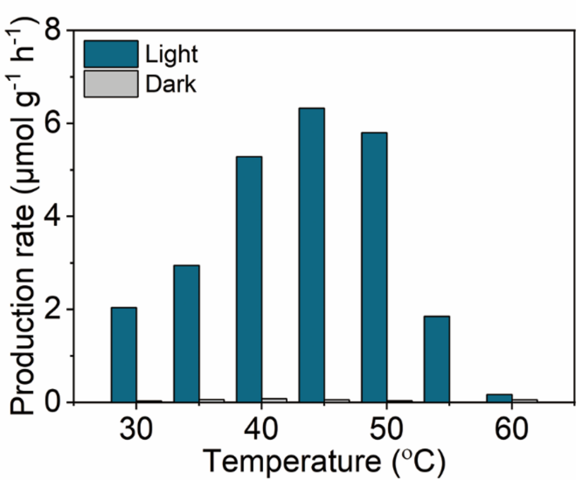

Fig. S25. Average production rates of CH_4 over Au rod@$CuPd_2$ under 800 nm light illumination with constant intensity of 400 mW cm^{-2} (cyan bars) or in dark environment (gray bars) at various temperatures between 30–60 ℃ in CO_2-saturated water.

In the dark condition, no any CH_4 product has been detected, demonstrating that the thermal effect cannot initiate the CO_2RR (Fig. S25). In sharp contrast, under constant monochromatic light illumination (800 nm, 400 mW cm^{-2}), the production rates of CH_4 show a volcano-shaped relationship with the reaction temperature and reach the maximum at 45 °C.

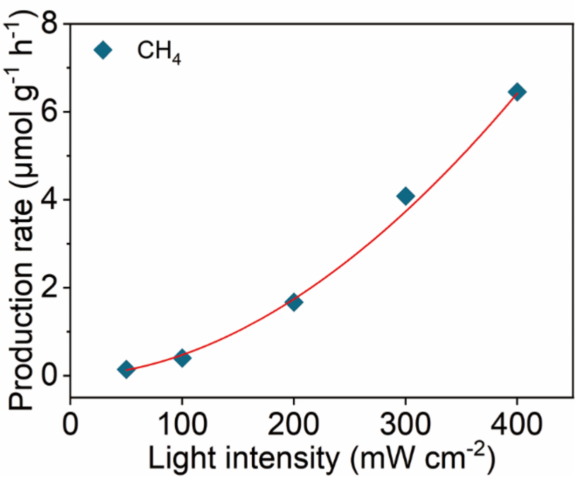

Fig. S26. Average production rates of CH_4 over Au rod@$CuPd_2$ at thermostatic control of 45 ℃ under 800 nm light illumination with an intensity range of 50–400 mW cm^{-2} in CO_2-saturated water.

After confirming the optimal temperature, we further perform CO_2RR by altering light intensity. The superlinear law dependence in Fig. S26 appears between CH_4 production rate and light intensity, suggesting that the CO_2RR using Au rod@$CuPd_2$ is a reaction involving multiple-photon excitation.

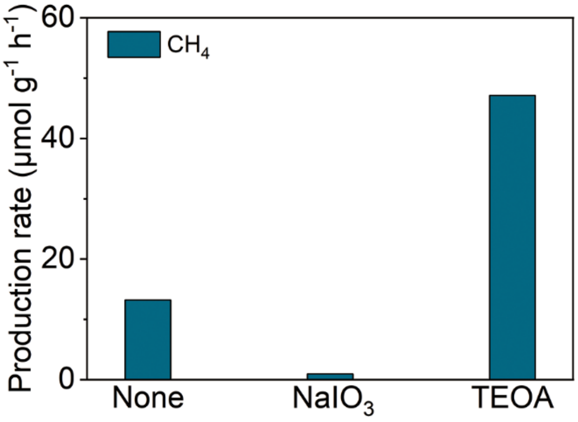

Fig. S27. Average production rates of CH_4 over Au rod@$CuPd_2$ under irradiation of 400 mW cm^{-2} full-spectrum light in CO_2-saturated water with no sacrificial agent (None) or sodium Iodate ($NaIO_3$) as electron sacrificial agent or triethanolamine (TEOA) as hole sacrificial agent.

In addition, the CO_2RR tests are also performed with the addition of electron or hole sacrificial agent, showing a decrease (0.9 μmol g^{-1} h^{-1}) and increase (47.1 μmol g^{-1} h^{-1}) in CH_4 production rate in comparison with that in the absence of sacrificial agent, respectively (Fig. S27). These results conclusively affirm the crucial role of plasmon-induced hot electrons in CO_2RR over Au rod@$CuPd_2$.

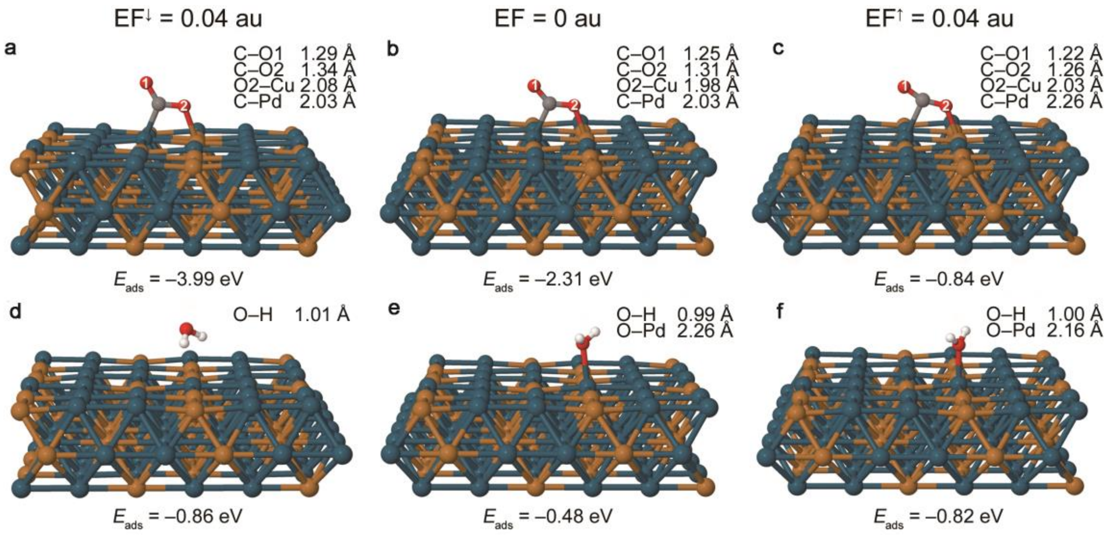

Fig. S28. (**a**–**f**) Optimization geometries of CO_2 (a–c) and H_2O (d–f) adsorbed on a CuPd (100) surface in the absence (middle column) and presence of electric field (left and right columns). The field pointing to the surface is denoted as EF$^{\downarrow}$ and away from the surface is represented as EF$^{\uparrow}$. The key bond lengths relevant to the adsorbed molecules and adsorption energies are listed in the individual structures.

The optimized geometries and adsorption energies of CO_2 adsorbed on CuPd (100) surface with and without electric field are illustrated in Fig. S28, a to c. When the applied electric field points to the surface, the C–O bond length of adsorbed CO_2 is elongated and the corresponding adsorption energy is reduced from –2.31 to –3.99 eV (Fig. S28b). In contrast, the opposite electric field leads to a shorter C–O bond length and a less stable adsorption state (–0.84 Å) (Fig. S28c). Nonetheless, the adsorption energy remains sufficiently negative to maintain the stable adsorption of CO_2 molecules. Under these circumstances, the adsorbed CO_2 molecules are forced to bend (electric field pointing toward sample) and stretch (electric field pointing away from the sample) with the high-frequency oscillation electric field, thereby being driven to the vibrational excited state. Therefore, when the electric field points toward the catalyst surface, the cleavage of the C–O bond can be triggered over a much lower energy barrier compared to the case in the absence of an electric field. In addition, we have performed a similar simulation by substituting H_2O for CO_2 molecules, showing that the interaction between H_2O and CuPd (100) surface is relatively weak, and thus the electric field can only slightly change the bond length of O–H (Fig. S28, d to f).

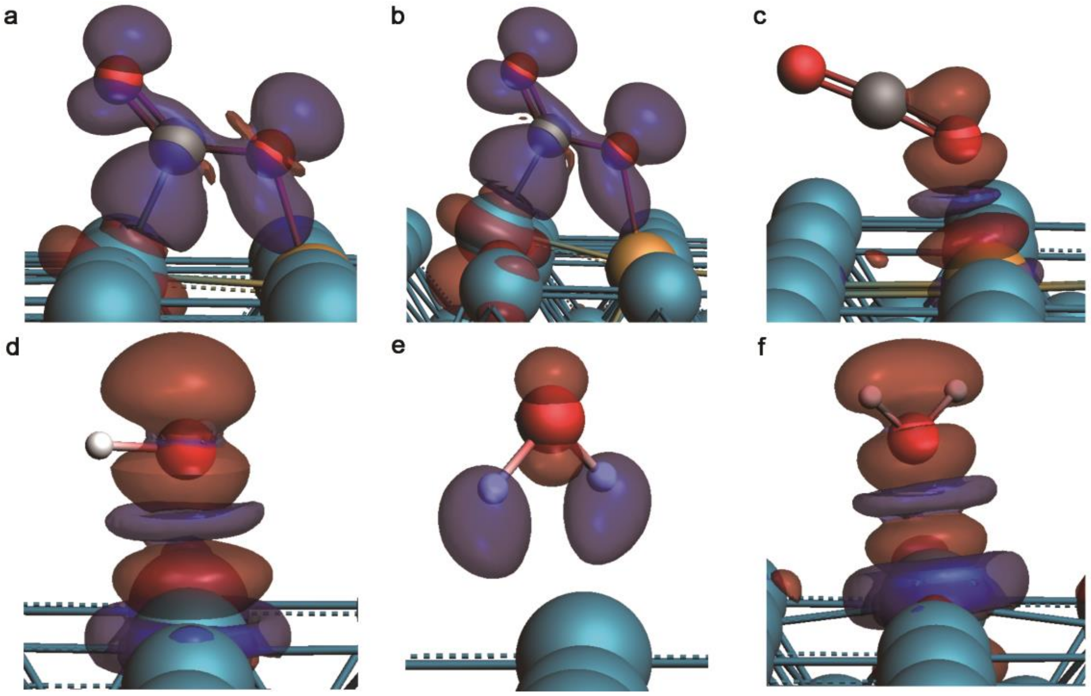

Fig. S29. (**a**–**f**) The most important deformation density from pEDA analysis with NOCV extension in CO_2 (a–c) and H_2O (d–f) adsorbed on a CuPd (100) surface in the absence (left column) and presence of electric field towards (middle column) or away from (right column) the surface. The color code indicates a charge flow from red to blue. Isovalue is set to 0.0005.

We explore how the charges flow between reactants and CuPd (100) surface. According to the simulation results in Fig. S29, the Hirshfeld charges are –0.136 e, –0.776 e and 0.154 e on the CO_2 molecules and –0.003 e, –0.346 e and 0.227 e on the H_2O molecules in the absence and presence of electric fields toward and away from the CuPd (100) surface, respectively. The charge transfer is from CO_2/H_2O to CuPd when the electric field points toward metal surface, and such a charge transfer reverses under the electric field pointing oppositely. It should be also noted that the Hirshfeld charge on the H_2O molecule is close to 0 in the absence of electric field (Fig. S29), implying that the electrons can hardly migrate to the H_2O. This result clearly shows that, although H_2O molecules are not as sensitive as CO_2 to the alternating electric field, the effect of the plasmon-induced electric field can also play an indispensable role in facilitating the migration of electrons from catalyst to H_2O for the subsequent reduction reaction.

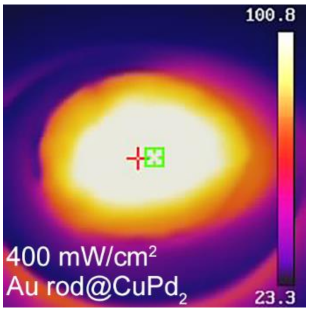

Fig. S30. Surface thermography of Au rod@$CuPd_2$ on a quartz wool sheet under 400 mW cm^{-2} full-spectrum light illumination.

In order to assess the local temperature on the surface of the nanoparticles, we employ the infrared thermal imager to obtain thermal profile of our catalyst sample under 400 mW cm^{-2} full-spectrum light illumination. As shown in Fig. S30, the highest temperature can reach 100.8 ℃ at the center of the quartz wool sheet after 5 min illumination.

Table S1. The molar ratios of Pd/Cu and CuPd/Au in the Au rod@$CuPd_2$-*y* samples calculated from different precursor usages and experimentally determined by ICP-MS.

Sample	Experimental molar ratio of Pd/Cu	Calculated molar ratio of Pd/Cu	Experimental molar ratio of CuPd/Au	Calculated molar ratio of CuPd/Au
Au rod@$CuPd_2$-0.1	2.6	1.0	0.1	0.2
Au rod@$CuPd_2$-0.2	2.4	1.0	0.2	0.5
Au rod@$CuPd_2$-0.6	2.0	1.0	0.6	1.0
Au rod@$CuPd_2$-0.9	1.9	1.0	0.9	1.5
Au rod@$CuPd_2$-1.2	2.2	1.0	1.2	2.0

The samples are denoted by Au rod@$CuPd_x$-y, where x is the molar ratio of Pd/Cu and y is the molar ratio of CuPd/Au. Given that the size of Au is fixed, the molar ratio of CuPd/Au can reflect the thickness of the shell.

Table S2. The molar ratios of Pd/Cu and CuPd/Au in the Au rod@CuPd$_x$ samples calculated from different precursor ratios and experimentally determined by ICP-MS.

Sample	Experimental molar ratio of Pd/Cu	Calculated molar ratio of Pd/Cu	Experimental molar ratio of CuPd/Au	Calculated molar ratio of CuPd/Au
Au rod@CuPd$_2$	2.0	1.0	0.6	1.0
Au rod@CuPd$_{2.5}$	2.5	1.5	0.6	1.0
Au rod@CuPd$_{3.6}$	3.6	2.3	0.6	1.0
Au rod@CuPd$_{6.1}$	6.1	4.0	0.7	1.0
Au rod@CuPd$_{8.8}$	8.8	9.0	0.7	1.0
Au rod@CuPd$_{14.2}$	14.2	19.0	0.7	1.0

For the sake of easy interpretation, the molar ratio of CuPd/Au is omitted when it is equal to 0.6 or 0.7.

Table S3. Fitting results of Pd K-edge and Cu K-edge EXAFS data.

Sample	Bond	CN	Bond length(Å)	$\sigma^2(10^{-3}$ Å$^2)$
Au rod@$CuPd_2$	Cu–Cu	1.6	2.58	10.9
	Cu–Pd	5.7	2.65	11.4
	Pd–Cu	2.1	2.65	11.4
	Pd–Pd	6.7	2.72	7.8
Au rod@$CuPd_{6.1}$	Cu–Cu	0.7	2.58	9.3
	Cu–Pd	6.6	2.68	9.0
	Pd–Cu	1.3	2.68	9.0
	Pd–Pd	8.6	2.73	7.6
Cu foil	Cu–Cu	12	2.54	7.9
Pd foil	Pd–Pd	12	2.74	5.9

Table S4. CH_4 production rates of plasmon-induced CO_2RR over Au rod@$CuPd_2$ under various reaction conditions.

Entry	Catalyst	Light [a]	Atmos-phere	Additional agent	Additional tempera-ture control	CH_4 production rate (μmol g^{-1} h^{-1})
1	× [b]	○ [c]	CO_2	×	×	Trace
2	○	×	CO_2	×	×	Trace
3	○	○	Ar	×	×	Trace
4	○	○	CO_2	– [d]	×	Trace
5	○	○	Ar	0.1 mM CTAB	×	Trace
6	○	○	Ar	0.1 mM CTAC	×	Trace
7	○	○	Ar	0.1 mM AA	×	Trace
8	×	○	Ar	0.1 mM CTAB	90 ℃	Trace
9	×	○	Ar	0.1 mM CTAC	90 ℃	Trace
10	×	○	Ar	0.1 mM AA	90 ℃	Trace
11	○	○	CO_2	0.1 mM CTAB	×	0.07
12	○	○	CO_2	0.1 mM CTAC	×	0.04
13	○	○	CO_2	0.1 mM AA	×	3.22
14	○	○	Ar	×	45 ℃	Trace

[a] This experiment is conducted under 400 mW cm^{-2} 800 nm light illumination.

[b] "×" stands for the case without catalyst or light illumination or additional temperature control.

[c] "○" stand for the case with catalyst or light illumination.

[d] "–" stand for the case without H_2O.

Table S5. CH_4 production rates of plasmon-induced CO_2RR over Au rod@$CuPd_2$ under various reaction conditions in a gas-solid biphase reactor.

Entry	Catalyst	Light [a]	Atmosphere	Additional agent	Additional temperature control	CH_4 production rate (μmol g^{-1} h^{-1})
1	○ [b]	× [c]	CO_2 + H_2O	×	100 ℃	Trace
2	○	×	CO_2 + H_2O	×	150 ℃	Trace
3	○	×	CO_2 + H_2O	×	200 ℃	Trace

[a] This experiment is conducted under 400 mW cm^{-2} light illumination.

[b] "×" stands for the case without catalyst or light illumination or additional temperature control.

[c] "○" stand for the case with catalyst or light illumination.

Considering that the actual surface temperature of the plasmonic metal nanostructures may be higher than the surface temperature detected by the thermal imager (see Fig. S30), we conduct the supplementary control experiments in the dark at 100, 150 and 200 ℃ in a mixture of CO_2 and H_2O vapor. Generally, the CH_4 can hardly be detected, indicating that the light is necessary for the production of CH_4.

Table S6. Comparison of the production rates toward artificial photosynthesis without using sacrificial agent.

Photocatalytic system	Primary product	Production rate ($\mu mol\ g^{-1}\ h^{-1}$)	Ref.
Cu/carbon nitride	CO	11.21	3
	CH_3OH	1.75	
	CH_4	0.61	
α-Fe_2O_3/g-C_3N_4	CO	27.2	4
ZnSe/CdS dot-on-rods	CO	11.3	5
NH_2-MIL-125/TiO_2@COF-366-Ni-OH-HAc	CO	16.87	6
Pt@h-BN	CH_4	9.2	7
TiO_2/conjugated porous polymers	CO	9	8
	CH_4	3	
TiO_2-mesocrystals/WO_{3-x}-nanowires	CH_4	16.3	9
CdS:Dy^{3+}/g-C_3N_4	CO	23.4	10
	CH_4	8.06	
FeTCP-OH-Co	CO	17.72	11
NNU-31-Zn	HCOOH	26.3	12
TTCOF-Zn	CO	4.48	13
Pt-defective CN	CH_4	6.3	14
C dot/O-modified CN	CH_3OH	24.2	15

Table S6. Continued.

Photocatalytic system	Primary product	Production rate (μmol g^{-1} h^{-1})	Ref.
$Cu_2O@Cu_3(BTC)_2$	CH_4	0.09	16
$MAPbI_3@PCN\text{-}221(Fe_{0.2})$	CO	6.64	17
	CH_4	12.85	
PFC-58-30	CO	7.27	18
	HCOOH	29.8	
COF-318-TiO_2	CO	69.7	19
$Bi_{19}Br_3S_{27}$	CH_3OH	0.88	20
	CO	0.5	
	CH_4	1.15	
TCOF-$MnMo_6$	CO	37.25	21
$WO_3{\cdot}ODDIN_2O$	CH_3COOH	9.4	22
$SiC@MoS_2$	CH_4	13.18	23
$Rb_{0.33}WO_3$	HCOOH	3.26	24
	CH_3OH	11.84	
BiOCl with Bi vacancies	CO	21.99	25
C-doped SnS_2	CH_3CHO	9.66	26
Ni-nanocluster loaded black TiO_2	CH_3CHO	1.6	27

Table S6. Continued.

Photocatalytic system	Primary product	Production rate (μmol g^{-1} h^{-1})	Ref.
Ag/TiO_2	CH_4	2.4	28
	CH_3OH	1.8	
	CO	1.3	
vacancy-defect $AgInP_2S_6$	C_2H_4	7.38	29
	CO	1.82	
	CH_4	0.93	
TiO_2/NH_2–UiO-66	CO	0.85	30
$CsPbBr_3$/USGO/α-Fe_2O_3	CO	73.8	31
Cu_2O/PCN	CH_3OH	34.5	32
$Cs_3Sb_2(Br_xI_{1-x})9$ ($0 \leqslant x \leqslant 1$) perovskite	CO	9.23	33
CoPcPDA-CMP	CO	14.27	34
Au rod@$CuPd_2$	CH_4	550	This work

Table S7. Comparison of the AQEs toward artificial photosynthesis without using sacrificial agent.

Photocatalytic system	Primary product	Catalyst type	Light irradiation wavelength (nm)	AQE (%)	Ref.
Cu/carbon nitride	$CO/MeOH/CH_4$	SC	353	1.32	3
Cd/N-doped graphene	CO/CH_4	SC	420	0.9	35
oxygen vacancy modified $BiOIO_3$	CO	SC	365	0.34	36
α-Fe_2O_3/g-C_3N_4	CO	SC	365	0.499	4
			420	0.963	
g-C_3N_4/MnO_x/Au-TiO_2	CH_4	SC	420	4.92	37
Ni single atom/ZrO_2	CO	SC	365	0.92	38
			420	0.36	
$BiOIO_3$ with {010} and {100} facets	CO	SC	365	0.1	39
TiO_2/graphdiyne	CO/CH_4	SC	365	0.2	40
$BiVO_4$ {010}–Au–Cu_2O	CH_4/CO	SC	500	0.44	41
$BiVO_4$ nanosheets/Zinc phthalocyanine	CO/CH_4	SC/homo	590	0.35	42
(cyclopentadienyl ruthenium)$_{0.6}$/TiO_2	CH_4	SC/homo	420	0.56	43
$Bi_{12}O_{17}Cl_2$	CO	SC	400	0.14	44
hierarchical treated rapepollen	CO	Biomaterial	420	6.7	45
PCN-601	CH_4/CO	MOF	504	2.18	46
$InVO_4$	CO	SC	385	0.54	47

Table S7. Continued.

Photocatalytic system	Primary product	Catalyst type	Light irradiation wavelength (nm)	AQE (%)	Ref.
V-defective orthorhombic $BiVO_4$	MeOH	SC	650	0.65	48
			350	5.96	
$CuInS_2$/ZnS/FeTPP	CO	QD/homo	450	0.01	49
Co_3O_4 hollow multi-shelled structures	CO	SC	350	0.017	50
			650	0.009	
SiC@MoS_2	CH_4	SC	400	1.75	23
			500	1.25	
$CsPbBr_3$/graphene oxide	CO/CH_4/H_2	Perovskite	400	0.025	51
O-defective WO_3	CO	SC	800	0.0274	52
treated polyheptazineimide /rGO	CH_4/CO/MeOH/ EtOH	SC	420	0.254	53
F-Pt-TiO_{2-x} mesoporous single crystals	CH_4	SC	350	0.038	54
ZnO-Cu_2O	CH_4	SC	370	1.5	55
C-doped SnS_2	CH_3CHO	SC	400	1.64	26
			600	0.32	
TiO_2-in-MIL-101-Cr-NO_2	CO/CH_4	SC/MOF	350	11	56
$Cs_2AgBiBr_6$	CO/CH_4	Perovskite	398	0.028	57

Table S7. Continued.

Photocatalytic system	Primary product	Catalyst type	Light irradiation wavelength (nm)	AQE (%)	Ref.
Au rod@$CuPd_2$	CH_4	LSPR	800	0.38	This work

SC: Semiconductor; Homo: Homogeneous photocatalyst; MOF: Metal organic frameworks; LSPR: Localized surface plasmon resonance catalyst

Supplementary References

1. Nikoobakht, B. & El-Sayed, M. A. Preparation and growth mechanism of gold nanorods (NRs) using seed-mediated growth method. *Chem. Mater.* **15**, 1957–1962 (2003).
2. Biesinger, M. C., Lau, L. W. M., Gerson, A. R. & Smart, R. S. C. Resolving surface chemical states in XPS analysis of first row transition metals, oxides and hydroxides: Sc, Ti, V, Cu and Zn. *Appl. Surf. Sci.* **257**, 887–898 (2010).
3. Wang, J. *et al.* A single Cu-center containing enzyme-mimic enabling full photosynthesis under CO_2 reduction. *ACS Nano* **14**, 8584–8593 (2020).
4. Jiang, Z. *et al.* A hierarchical Z-scheme α-Fe_2O_3/g-C_3N_4 hybrid for enhanced photocatalytic CO_2 reduction. *Adv. Mater.* **30**, 1706108 (2018).
5. Xin, Z. K. *et al.* Rational design of dot-on-rod nano-heterostructure for photocatalytic CO_2 reduction: pivotal role of hole transfer and utilization. *Adv. Mater.* **34**, e2106662 (2022).
6. Zhang, M. *et al.* Controllable synthesis of COFs-based multicomponent nanocomposites from core-shell to yolk-shell and hollow-sphere structure for artificial photosynthesis. *Adv. Mater.* **33**, e2105002 (2021).
7. Bi, W., Hu, Y., Jiang, H., Zhang, L. & Li, C. Revealing the sudden alternation in Pt@h-BN nanoreactors for nearly 100% CO_2-to-CH_4 photoreduction. *Adv. Funct. Mater.* **31**, 2010780 (2021).
8. Collado, L. *et al.* Conjugated porous polymers based on BODIPY and BOPHY dyes in hybrid heterojunctions for artificial photosynthesis. *Adv. Funct. Mater.* **31**, 2105384 (2021).
9. Lou, Z. *et al.* Plasmonic heterostructure TiO_2-MCs/WO_{3-x}-NWs with continuous photoelectron injection boosting hot electron for methane generation. *Adv. Funct. Mater.* **29**, 1808696 (2019).
10. Zhao, Y. *et al.* Dual functions of CO_2 molecular activation and 4*f* levels as electron transport bridge in dysprosium single atom composite photocatalysts with enhanced visible light photoactivities. *Adv. Funct. Mater.* **31**, 2104976 (2021).
11. Chen, E. X. *et al.* Energy band alignment and redox-active sites in metalloporphyrin-spaced metal-catechol frameworks for enhanced CO_2 photoreduction. *Angew. Chem. Int. Ed.* **61**, e202111622 (2022).
12. Dong, L. Z. *et al.* Stable heterometallic cluster-based organic framework catalysts for artificial photosynthesis. *Angew. Chem. Int. Ed.* **59**, 2659–2663 (2020).
13. Lu, M. *et al.* Rational design of crystalline covalent organic frameworks for efficient CO_2 photoreduction with H_2O. *Angew. Chem. Int. Ed.* **58**, 12392–12397 (2019).
14. Shi, X. *et al.* Highly selective photocatalytic CO_2 methanation with water vapor on single-atom platinum-decorated defective carbon nitride. *Angew. Chem. Int. Ed.* **61**, e202203063 (2022).
15. Wang, Y., Godin, R., Durrant, J. R. & Tang, J. Efficient hole trapping in carbon dot/oxygen-modified carbon nitride heterojunction photocatalysts for enhanced methanol production from CO_2 under neutral conditions. *Angew. Chem. Int. Ed.* **60**, 20811–20816 (2021).
16. Wu, H. *et al.* Metal-organic framework decorated cuprous oxide nanowires for long-lived charges applied in selective photocatalytic CO_2 reduction to CH_4. *Angew. Chem. Int. Ed.* **60**, 8455–8459 (2021).
17. Wu, L. Y. *et al.* Encapsulating perovskite quantum dots in iron-based metal-organic frameworks (MOFs) for efficient photocatalytic CO_2 reduction. *Angew. Chem. Int. Ed.* **58**, 9491–9495 (2019).
18. Zhang, A. A. *et al.* Partial metalation of porphyrin moieties in hydrogen-bonded organic frameworks provides enhanced CO_2 photoreduction activity. *Angew. Chem. Int. Ed.* **134**,

e202203955 (2022).
19. Zhang, M. *et al.* Semiconductor/covalent-organic-framework Z-scheme heterojunctions for artificial photosynthesis. *Angew. Chem. Int. Ed.* **59**, 6500–6506 (2020).
20. Li, J. *et al.* Interfacial engineering of $Bi_{19}Br_3S_{27}$ nanowires promotes metallic photocatalytic CO_2 reduction activity under near-infrared light irradiation. *J. Am. Chem. Soc.* **143**, 6551–6559 (2021).
21. Lu, M. *et al.* Confining and highly dispersing single polyoxometalate clusters in covalent organic frameworks by covalent linkages for CO_2 photoreduction. *J. Am. Chem. Soc.* **144**, 1861–1871 (2022).
22. Sun, S., Watanabe, M., Wu, J., An, Q. & Ishihara, T. Ultrathin $WO_3 \cdot 0.33H_2O$ nanotubes for CO_2 photoreduction to acetate with high selectivity. *J. Am. Chem. Soc.* **140**, 6474–6482 (2018).
23. Wang, Y. *et al.* Visible-light driven overall conversion of CO_2 and H_2O to CH_4 and O_2 on 3D-SiC@2D-MoS_2 heterostructure. *J. Am. Chem. Soc.* **140**, 14595–14598 (2018).
24. Wu, X. *et al.* Photocatalytic CO_2 conversion of $M_{0.33}WO_3$ directly from the air with high selectivity: insight into full spectrum-induced reaction mechanism. *J. Am. Chem. Soc.* **141**, 5267–5274 (2019).
25. Wang, L. *et al.* Bismuth vacancy-induced efficient CO_2 photoreduction in BiOCl directly from natural air: a progressive step toward photosynthesis in nature. *Nano Lett.* **21**, 10260–10266 (2021).
26. Shown, I. *et al.* Carbon-doped SnS_2 nanostructure as a high-efficiency solar fuel catalyst under visible light. *Nat. Commun.* **9**, 169 (2018).
27. Billo, T. *et al.* Ni-nanocluster modified black TiO_2 with dual active sites for selective photocatalytic CO_2 reduction. *Small* **14**, 1702928 (2018).
28. Collado, L. *et al.* Unravelling the effect of charge dynamics at the plasmonic metal/semiconductor interface for CO_2 photoreduction. *Nat. Commun.* **9**, 4986 (2018).
29. Gao, W. *et al.* Vacancy-defect modulated pathway of photoreduction of CO_2 on single atomically thin $AgInP_2S_6$ sheets into olefiant gas. *Nat. Commun.* **12**, 4747 (2021).
30. Crake, A. *et al.* The effect of materials architecture in TiO_2/MOF composites on CO_2 photoreduction and charge transfer. *Small* **15**, e1805473 (2019).
31. Mu, Y. F. *et al.* Ultrathin and small-size graphene oxide as an electron mediator for perovskite-based Z-scheme system to significantly enhance photocatalytic CO_2 reduction. *Small* **16**, e2002140 (2020).
32. Tian, Z. Y. *et al.* Construction of low-cost Z-scheme heterostructure Cu_2O/PCN for highly selective CO_2 photoreduction to methanol with water oxidation. *Small* **17**, e2103558 (2021).
33. Wu, D. *et al.* Synthesis of stable lead-free $Cs_3Sb_2(Br_xI_{1-x})_9$ ($0 \leqslant x \leqslant 1$) perovskite nanoplatelets and their application in CO_2 photocatalytic reduction. *Small* **18**, e2106001 (2022).
34. Zhi, Q. *et al.* Covalent microporous polymer nanosheets for efficient photocatalytic CO_2 conversion with H_2O. *Small* **18**, e2201314 (2022).
35. Bie, C., Zhu, B., Xu, F., Zhang, L. & Yu, J. In situ grown monolayer N-doped graphene on CdS hollow spheres with seamless contact for photocatalytic CO_2 reduction. *Adv. Mater.* **31**, e1902868 (2019).
36. Chen, F. *et al.* Macroscopic spontaneous polarization and surface oxygen vacancies collaboratively boosting CO_2 photoreduction on $BiOIO_3$ single crystals. *Adv. Mater.* **32**, e1908350 (2020).
37. Raziq, F. *et al.* Synthesis of large surface-area g-C_3N_4 comodified with MnO_x and Au-TiO_2 as efficient visible-light photocatalysts for fuel production. *Adv. Energy Mater.* **8**, 1701580 (2018).

38. Xiong, X. Y. *et al.* Photocatalytic CO_2 reduction to CO over Ni single atoms supported on defect-rich zirconia. *Adv. Energy Mater.* **10**, 2002928 (2020).
39. Chen, F. *et al.* Thickness-dependent facet junction control of layered $BiOIO_3$ single crystals for highly efficient CO_2 photoreduction. *Adv. Funct. Mater.* **28**, 1804284 (2018).
40. Xu, F. *et al.* Graphdiyne: a new photocatalytic CO_2 reduction cocatalyst. *Adv. Funct. Mater.* **29**, 1904256 (2019).
41. Zhou, C. *et al.* A facet-dependent schottky-junction electron shuttle in a $BiVO_4$\{010\}-Au-Cu_2O Z-scheme photocatalyst for efficient charge separation. *Adv. Funct. Mater.* **28**, 1801214 (2018).
42. Bian, J. *et al.* Dimension-matched zinc phthalocyanine/$BiVO_4$ ultrathin nanocomposites for CO_2 reduction as efficient wide-visible-light-driven photocatalysts via a cascade charge transfer. *Angew. Chem. Int. Ed.* **58**, 10873–10878 (2019).
43. Huang, H. *et al.* A long-lived mononuclear cyclopentadienyl ruthenium complex grafted onto anatase TiO_2 for efficient CO_2 photoreduction. *Angew. Chem. Int. Ed.* **55**, 8314–8318 (2016).
44. Di, J. *et al.* Defect-rich $Bi_{12}O_{17}Cl_2$ nanotubes self-accelerating charge separation for boosting photocatalytic CO_2 reduction. *Angew. Chem. Int. Ed.* **57**, 14847–14851 (2018).
45. Jiang, Z. *et al.* Nature-based catalyst for visible-light-driven photocatalytic CO_2 reduction. *Energy Environ. Sci.* **11**, 2382–2389 (2018).
46. Fang, Z. B. *et al.* Boosting interfacial charge-transfer kinetics for efficient overall CO_2 photoreduction via rational design of coordination spheres on metal-organic frameworks. *J. Am. Chem. Soc.* **142**, 12515–12523 (2020).
47. Han, Q. *et al.* Convincing synthesis of atomically thin, single-crystalline $InVO_4$ sheets toward promoting highly selective and efficient solar conversion of CO_2 into CO. *J. Am. Chem. Soc.* **141**, 4209–4213 (2019).
48. Gao, S. *et al.* Highly efficient and exceptionally durable CO_2 photoreduction to methanol over freestanding defective single-unit-cell bismuth vanadate layers. *J. Am. Chem. Soc.* **139**, 3438–3445 (2017).
49. Lian, S., Kodaimati, M. S., Dolzhnikov, D. S., Calzada, R. & Weiss, E. A. Powering a CO_2 reduction catalyst with visible light through multiple sub-picosecond electron transfers from a quantum dot. *J. Am. Chem. Soc.* **139**, 8931–8938 (2017).
50. Wang, L., Wan, J., Zhao, Y., Yang, N. & Wang, D. Hollow multi-shelled structures of Co_3O_4 dodecahedron with unique crystal orientation for enhanced photocatalytic CO_2 reduction. *J. Am. Chem. Soc.* **141**, 2238–2241 (2019).
51. Xu, Y. F. *et al.* A $CsPbBr_3$ perovskite quantum dot/graphene oxide composite for photocatalytic CO_2 reduction. *J. Am. Chem. Soc.* **139**, 5660–5663 (2017).
52. Liang, L. *et al.* Infrared light-driven CO_2 overall splitting at room temperature. *Joule* **2**, 1004–1016 (2018).
53. Xia, Y. *et al.* Highly selective CO_2 capture and its direct photochemical conversion on ordered 2D/1D heterojunctions. *Joule* **3**, 2792–2805 (2019).
54. Xing, M. *et al.* Modulation of the reduction potential of TiO_{2-x} by fluorination for efficient and selective CH_4 generation from CO_2 photoreduction. *Nano Lett.* **18**, 3384–3390 (2018).
55. Bae, K. L., Kim, J., Lim, C. K., Nam, K. M. & Song, H. Colloidal zinc oxide-copper(I) oxide nanocatalysts for selective aqueous photocatalytic carbon dioxide conversion into methane. *Nat. Commun.* **8**, 1156 (2017).
56. Jiang, Z. *et al.* Filling metal-organic framework mesopores with TiO_2 for CO_2 photoreduction. *Nature* **586**, 549–554 (2020).

57. Zhou, L., Xu, Y. F., Chen, B. X., Kuang, D. B. & Su, C. Y. Synthesis and photocatalytic application of stable lead-free $Cs_2AgBiBr_6$ perovskite nanocrystals. *Small* **14**, e1703762 (2018)
